# The multipurpose cell factory *Aspergillus niger* can be engineered to produce hydroxylated collagen

**DOI:** 10.1186/s13068-025-02681-y

**Published:** 2025-08-08

**Authors:** Tom Morris, Friederike Gerstl, Sascha Jung, Timothy C. Cairns, Vera Meyer

**Affiliations:** Chair of Applied and Molecular Microbiology, Institute of Biotechnology, Technische Universität Berlin, Berlin, Germany

**Keywords:** *Aspergillus niger*, Collagen, Heterologous expression system, Transcriptomics

## Abstract

Advances in tissue printing and wound healing necessitate a continuous global supply of collagen. Microbial systems are highly desirable to meet these demands as recombinant collagenous proteins can be guaranteed as free from animal viruses. The filamentous cell factory *Aspergillus niger* has been instrumental for decades in the production of organic acids, enzymes and proteins, yet this fungus has not been explored for recombinant collagen production. In this study, we conducted extensive genetic engineering and fermentation optimization to provide proof of principle that *A. niger* can produce hydroxylated collagen. We used a modular cloning system to generate a suite of cassettes encoding numerous N-terminal secretion signals, native collagen genes and, additionally, various prolyl-4-hydroxylases (P4H) for protein hydroxylation. Collagen transcription was supported by both luciferase reporter and eGFP tagged approaches. Peptide sequencing from culture supernatant confirmed *A. niger* produced partially hydroxylated collagen. We then conducted a range of media optimizations and RNA sequencing to, respectively, increase collagen production and identify proteases which we hypothesized were detrimental to recombinant protein titers. Thus, we deleted an endopeptidase encoding gene, *protA*, which was likely responsible for degrading secreting collagen. Ultimately, we were able to generate an isolate capable of producing hydroxylated collagen at titers of 5 mgL^−1^ in shake flask models of fermentation. This study thus proves *A. niger* is a promising heterologous system to address the demand for virus-free collagen.

**Supplementary Information:**

The online version contains supplementary material available at 10.1186/s13068-025-02681-y.

## Background

Filamentous fungi play a crucial role within ecosystems as recyclers, primarily due to their ability to secrete a multitude of enzymes which degrade large polymers prior to osmotrophic uptake [1]. The filamentous ascomycete *Aspergillus niger,* with its naturally large secretion capabilities, has been utilized for the last century as a prolific producer of organic acids, with the global market expected to reach over 4 billion dollars by 2027 for citric and gluconic acid alone [2]. Alongside organic acids, *A. niger* currently boasts an impressive product portfolio of proteins, enzymes and secondary metabolites [3–5]. Importantly, *A. niger* and other fungi are able to enact an extensive range of post-translational modifications (PTMs) including but not limited to glycosylation, ubiquitination, or hydroxylation, which facilitate proper folding and function of recombinant proteins. However, this multipurpose cell factory has thus far not been explored for the production of fibrillar proteins such as collagen.

Collagen is one of the most abundant proteins in vertebrate organisms [6], and there are currently 28 identified collagen subtypes [7]. Encoded as procollagens, these native proteins subsequently form homo- or heterotrimers, leading to the formation of a characteristic triple helical formation. Finally, supramolecular structures of self-forming helices ultimately produce fibrillar collagen [8]. Collagen displays a unique amino acid sequence which is characterized by high proline and glycine content through a tripartite Gly-X–Y repeat, where X is any amino acid and Y is often hydroxyproline; a PTM to the amino acid proline [9]. The enzyme that catalyses the formation of hydroxyproline is prolyl 4-hydroxylase (P4H), which actively hydroxylates procollagen polypeptides in the lumen of the endoplasmic reticulum [10]. These enzymes are found throughout many eukaryotes, and require α-ketoglutarate, iron, ascorbate and oxygen for their functionality [11]. P4Hs undergo oxidative inactivation after performing hydroxylation and have been shown to remain inactive until reduction of their iron core by ascorbate which restores their catalytic potential [12].

The global collagen market reached approximately 4.7 billion dollars in 2020, the majority of which belonged to the food and beverage or health management industries [13]. Recent advancements in human tissue engineering and wound healing demand a collagen source entirely void of animal borne viruses that current purification processes from animal sources cannot provide. These animal derived collagens have also been linked to undesirable patient immunogenic reactions or potential disease transmission [14, 15]. Accordingly, there has been a recent drive towards the production of functional recombinant collagen-based biomaterials due to their high biocompatibility [16]. A study recently described that increasing the ratio of recombinant collagen III to gelatin (hydrolysed native collagen) resulted in reduced immunoreactions and increased wound healing [17]. However, current recombinant collagen systems still display the disadvantage of high cost and limited yield ratio. Yeasts such as *Pichia pastoris* often require methanol for induction of gene expression which is unfavourable for human applications [18] and plant systems produce low yields and have difficulties with downstream purifications [19]. Bacterial systems are generally incapable of the required PTMs and mammalian cell lines present prohibitive costs due to required media components [20]. *A niger*, conversely, possesses high secretion capabilities for facile downstream purification, PTM capabilities for proline hydroxylation, an ever-growing molecular toolkit for recombinant protein expression and the ability for robust growth on a multitude of complex and economically viable carbon and nitrogen sources [5]. Likewise, this filamentous cell factory already holds a GRAS status allowing the use of recombinant products for a range of markets. In this study, we provide proof-of-principle that hydroxylated collagen can be produced in the multipurpose cell factory *A. niger*. Additionally, we generate evidence that expression of a viral P4H is able to hydroxylate collagen, and identify a putative protease encoding gene *protA* which likely is responsible for product degradation*.* Taken together, the data generated in this study confirm *A. niger* is a promising cell factory to address the global demand for recombinant collagen. Finally, the strains we have constructed constitute a starting point for further genetic engineering or bioprocess optimization to maximize yields in the near future.

## Methods and materials

### Strains and cultivations

The strains used and produced in this study are listed in Table 1. The progenitor isolate Δ*gaaB-Mg* was obtained from Peter Richards and is detailed in literature [21]. *Escherichia coli* TOP10 (Thermo Fisher Scientific, USA) was used for plasmid construction with the Modular Cloning system [22] and cultured at 37 °C in Luria–Bertani broth containing either ampicillin (100 µg mL^−1^), spectinomycin (100 µg mL^−1^) or kanamycin (50 µg mL^−1^).

**Table 1:** Strains used and produced within this study

Name	Genotype	Notes	Experimental use	Background isolates	References
*gaaB-Mg*	Δ*gaaB;* *PgpdA-MgGADH; pyrG* ^+^	Progenitor strain for mutant construction expressing recombinant GalUA dehydratase from *Malpighia glabra*	Progenitor isolate	ATCC 1015	[21]
*gaaB-Mg*-17.2	Δ*gaaB; pyrG*^*−*^	*pyrG* mutant progenitor	Progenitor isolate	Δ*gaaB-Mg*	This study
VG8.27	Tet-On *mluc*, *pyrG* ^+^	Luciferase under Tet-on promoter	Luciferase control	AB4.1 (N402 derivative)	[35]
TM4.1, TM4.2	Tet-on-collagen III-P2A-luciferase; humanP4H*; pyrG*^+^	Inducible collagen and luciferase, human P4H	Luciferase assay	Δ*gaaB-Mg*-17.2	This study
TM5.3, TM5.5	Tet-on-collagen III-P2A-luciferase; plant P4H*; pyrG*^+^	Inducible collagen and luciferase, plant P4H	Luciferase assay	Δ*gaaB-Mg*-17.2	This study
TM6.1, TM6.2	Tet-on-collagen III-P2A-luciferase; viral P4H*; pyrG*^+^	Inducible collagen and luciferase, viral P4H	Luciferase assay/SDS-PAGE	Δ*gaaB-Mg*-17.2	This study
FGE2.1	Δ*gaaB; PgpdA-MgGADH; pyrG *^*−*^*; prtT *^*−*^	Protease deficient progenitor	Progenitor isolate	Δ*gaaB-Mg*-17.2	This study
TM17.1	Tet-on-collagen III-eGFP*; pyrG* ^+^	Inducible eGFP tagged Collagen III, viral P4H	Microscopy	FGE2.1	This study
TM4.2.1	Tet-on-collagen III-P2A-luciferase*; pyrG*^+^	*prtT*^−^ collagen III Human P4H	SDS-PAGE	FGE2.1	This study
TM20.1	Tet-on-*glaA*-SS-collagen III-HiBiT*; pyrG*^+^	HiBiT tagged collagen Human P4H	Cassette optimization	FGE2.1	This study
TM27.4	Tet-on-*pgxA*-SS-collagen III-HiBiT*; pyrG*^+^	HiBiT tagged collagen Human P4H	Cassette optimization	FGE2.1	This study
TM29.6	*Ptef1*-amylase SS-collagen III-HiBiT*; pyrG*^+^	HiBiT tagged collagen Human P4H	Cassette optimization	FGE2.1	This study
TM30.4	*Ptef1*-*pgxA*-SS-collagen III-HiBiT*; pyrG*^+^	HiBiT tagged collagen Human P4H	Cassette optimization	FGE2.1	This study
TM31.2	*Ptef1*-*pgaI*-SS-collagen III-HiBiT*; pyrG*^+^	HiBiT tagged collagen Human P4H	Cassette optimization	FGE2.1	This study
AT2.1	*prtT*^−^; *pyrG*^−^; ∆*protA*	∆*protA* protease deficient progenitor	Intermediate	FGE2.1	This study
AT6.4	*prtT* ^−^; *pyrG* ^−^; ∆*protA; PhttA-gaaA; Prpl15-gatA*	Intermediate progenitor strain with *gaaA* and *gatA* active in glucose replete conditions	Intermediate	FGE2.1	This study
TM39.8	*Ptef1-pgxA*-SS-collagen III-HiBiT*; pyrG*^+^	HiBiT tagged collagen Human P4H	Optimization	AT6.4	This study
TM43.4	*Ptef1-pgxA*-SS-collagen III-HiBiT*; pyrG*^+^	HiBiT tagged collagen Plant P4H	Optimization	AT6.4	This study
TM44.2	*Ptef1-pgxA*-SS-collagen III-HiBiT*; pyrG*^+^	HiBiT tagged collagen Viral P4H	Optimization	AT6.4	This study

*A. niger* was cultivated on defined minimal medium (MM) as described previously [23] or on complete medium (CM), where MM was supplemented with 0.5% yeast extract and 0.1% casamino acids. 1.5% agar was supplemented for growth on solid media. Conidia for inoculation were harvested from solidified CM using sterile 0.9% (w/v) NaCl solution. 0.75% 5-fluoroorotic acid (5-FOA) and 10 mM uridine was supplemented into MM to facilitate loss of the *pyrG* marker where necessary. GalUA medium was a glucose-free MM, supplemented with 20 g L^−1^ filter sterilized D-galacturonic acid, 5% xylose and 0.003% yeast extract [21].

### Genetic manipulation

Plasmids used in this study are listed in Additional File 1. Modular cloning plasmids are listed as pTM_X_Y where X denotes the level (0—ORFs, promoters, terminators; 1—full transcriptional unit with promoter and terminator; 2—multigene constructs). Secretion signals and protein tags were incorporated into the level 0 plasmids. Primers used are listed in Additional File 2. Pre-constructed plasmids from an in-house MoClo library are listed as pMC_X_Y. All level 0 plasmids were sequence verified by Sanger Sequencing (LGC Genomics, Berlin). All level 1 and level 2 plasmids were confirmed by restriction enzyme digest.

The 20 bp protospacers for Cas12a-mediated genome editing are highlighted in bold within the primers used and were designed using *A. niger* CBS513.88 genome annotations. Synthetic single stranded constructs containing a T7 promoter, the 20 bp target and a Cas12a recognition site were provided by (LGC Genomics, Berlin). DNA templates for in vitro sgRNA synthesis (MegaScript T7 Transcription Kit, Thermo Fisher Scientific, USA) were ligated using oligo extension PCR with an in-house oligo containing the reverse complement Cas12a recognition sequence. Donor DNA with micro-homology (60 bp) flanks were generated as described in literature [24], which were adjacent to but not containing the PAM site of the target sequence. Plasmids listed in Additional File 1 were used as the template to produce these donor DNA, which were purified using a PCR purification kit (innuPREP DOUBLEpure Kit, Analytik Jena).

### DNA transformation

CRISPR/Cas12a modifications were performed using the ribonucleoprotein (RNP) approach and a PEG-mediated transformation using 60% PEG 4000 [25]. During transformation, 2 µg donor DNA constructs containing the *pyrG* selection marker were co-transformed with 1 µL sgRNA and 20 µg purified Cas12a protein into the protoplasts of *A. niger* isolates used. The sgRNA was coupled to the Cas12a protein in vitro at 37 °C for 15 min in a 20 µL reaction with 2 µL Tango buffer (Thermo Fisher Scientific, USA) before use. After twice subculture and purification, genomic DNA of selected transformants was extracted and used for targeted integration verification via diagnostic PCR.

For constructs requiring curing of the *pyrG* marker, conidial suspensions of confirmed transformants were sub-cultured onto MM containing 5-FOA (50 µg/mL) and uridine (20 mM). Colonies capable of growth were presumed to no longer contain the *pyrG* sequence through a recombination event between the 5′ and 3′ *A. oryzae pyrG* terminator sequences due to selective pressure of the 5-FOA.

### Phenotypic analysis on solid media

To confirm the phenotype of the *pyrG* status of mutants, *A. niger* conidia were harvested from 5-day cultivated CM agar plates in sterile 0.9% NaCl (w/v). 10 µL spore solutions (10^5^ mL^−1^) were spotted on MM agar plates without uridine, with uridine or with 5-FOA and uridine and incubated for 4 days at 30 °C.

For protease-deficient mutants, *A. niger* conidia were harvested as above and spotted onto MM supplemented with 0.4% casein as previously described [26]. The absence of a clearance halo in the media was used as confirmation of proteolytic deficiency of the putative transformants.

### Transcriptome data analysis

Transcriptomic analysis was performed as published previously [27, 28]. Briefly, total RNA was isolated from biomass samples in TRIzol™ reagent (Thermo Fisher Scientific, USA) using the Direct-zol RNA MiniPrep Kit (Zymo). All samples were confirmed to have an A_260/280_ ratio larger than 2.0 for purity, and 15 µL purified total RNA containing 5–15 µg was sent for analysis to Azenta Life Sciences (USA) European office GENEWIZ (Leipzig, Germany). Illumina sequencing library preparation RNA with PolyA selection was performed with 10 M read pairs and raw data delivered in FASTQ format. *A. niger* N402 was used as the reference genome and mapping was performed with STAR version 2.7.10a [29] to produce bam files that were processed in R. RStudio workflow was based on R (version 4.2.3) and the R packages devtools (version 2.4.4), Rsamtools (version 2.12.0), GenomicFeatures (version 1.48.3), GenomicAlignments (version 1.26.1), and DESeq2 (version 1.36.0). GenomicAlignment was used to count genes (command: summarizeOverlaps) and DESeq was used to normalize gene counts (command: estimateSizeFactors). Multiple hypothesis corrected p-values were chosen to assess statistical significance. A unicate biological sample for the 21 h time point was used due to insufficient reads of the duplicate for DGE analysis. Transcriptomic data were deposited to the NCBI SRA database under PRJNA1229437.

### Luciferase luminescence

Strains containing Tet-on inducible collagen cassette were inoculated (5.0 × 10^6^ spores mL^−1^) to a 96-well plate into CM (200 µl) containing beetle luciferin (0.4 mM) and doxycycline (20 µg mL^−1^). Luminescence was detected over 24 h using a Perkin Elmer 2030 Multilabel Reader VictorTM X3 with the average of experimental triplicates normalized to OD at 595 nm depicted. Negative control (NC) was uninoculated media, positive control (PC) was VG8.27 expressing luciferase under the Tet-on promoter.

### Fluorescence microscopy

Conidial suspensions (10 µL, 1.0 × 10^5^ spores mL^−1^) were cultivated for 24 h at room temperature on solid minimal medium containing 20 µg/mL doxycycline and fluorescence was measured with excitation at 450–490 nm and emission at 500–550 nm with a differential interference microscope (Leica).

### SDS-PAGE

Culture filtrates (20 µL) mixed with 5X reducing buffer (5 µL), pH adjusted using 5 M NaOH (0.5–1 µL, until blue) and heated at 95 °C. Proteins from 20 µL of this solution, and PageRuler™ Pre-stained protein ladder (5 µL, Thermo Fisher Scientific, USA) were separated by electrophoresis at 120 V for approximately 1 h using pre-cast gradient gels (5–18%, Bio-Rad Laboratories, USA) using the Laemmli procedure (Laemmli, 1970). Staining was performed using Coomassie G (0.075%) in 35 mM HCl with warming for 1 h and destained with MQ. Bands for LC–MS identification were excised.

### HiBiT Western blot

No deviations were made from the Promega protocol for HiBiT detection. Briefly, proteins from SDS-PAGE experiments (unstained) were transferred to nitrocellulose membrane (0.2 µM, Thermo Fisher Scientific) using a semi-dry transfer method with Whatman® paper and 1xPTB in methanol (10 × PTB: 30.3 g L^−1^ Tris, 144.1 g L^−1^ glycine, 1% SDS, MQ) for 40 min at 20 V. The HiBiT tag was solubilized on the membrane using 1xTBST for 30 min (10 × TBST: 2.4 g L^−1^ Tris HCl, 8.8 g L^−1^ NaCl, 0.1% Tween 20). Detection of the HiBiT tag was achieved using a chemiluminescence (Bio-Rad, USA) with signal accumulation image overlay for 30 sec intervals for 2 min. For Dot Blot experiments, a 96-well format was undertaken using the Filtration Manifold Kit Schleicher and Schuell, Germany). 100 µL crude culture filtrate was heated for 5 min at 95 °C, centrifuged to remove precipitates, applied directly to a nitrocellulose membrane and detection of HiBiT tagged proteins performed as above.

## Results

### Construction of a suite of collagen expressing cassettes using modular cloning

The modular cloning system [22] has previously been used to generate a synthetic biology toolkit for a range of industrially used filamentous fungi [30]. This system enables rapid assembly of different cassettes derived from a variety of promoters, terminators, epitope tags, secretion signals, or transformation markers.

**Fig. 1:**
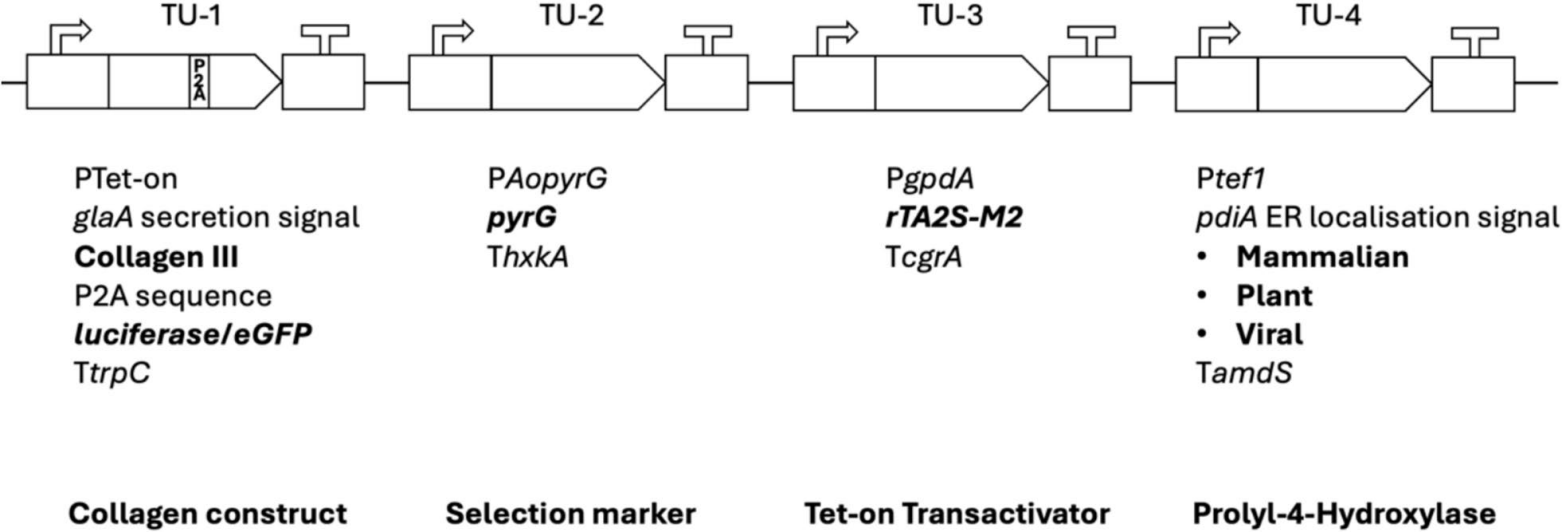
Modular cloning for the construction of a suite of collagen expressing cassettes. The modular cloning system allows interchangeable transcriptional units (TU) to be ligated together into a single plasmid backbone. TU-1 contains the collagen III sequence fused to the *glaA* secretion signal. Constructs utilized polycistronic expression of luciferase using a P2A sequence or an *eGFP* tagged collagen III. TU-4 contains the prolyl-4-hydroxylase variant in bold. Transcriptional units may also be replaced by „dummy “ units, 8 bp flanked by the 4 bp sticky ends for ligation that confer no biological function. All plasmids in this study were constructed with transcriptional units in this order. P: promoter; T: terminator

We aimed to generate a dedicated collagen modular cloning library for use in this study and future strain optimization efforts (Fig. 1). This method allowed high-throughput construction of a library of cassettes expressing a variety of promoters, signal peptides, reporters (luciferase/eGFP), and markers (Fig. 1 and Table 1). Key encoded proteins in the library include a fragment of COL3A1 from the wild boar *Sus scrofa* (NCBI: 100152001) and three P4H variants, including human (NCBI: alpha subunit 5033; beta subunit 5034), plant (*Arabidopsis thaliana,* NCBI: 818910) or viral (*Paramecium bursaria* Chlorella virus-1, NCBI: 917814). The P4H sequences chosen have been experimentally verified for recombinant collagen production and proline hydroxylation capabilities [31–33]. Coding sequences and modifications are given in Additional File 3.

We also encoded a P2A sequence for polycistronic expression which enables equimolar expression of collagen and luciferase under control of a single Tet-on doxycycline inducible promoter [34]. Variations of these transcriptional units (inducible or constitutive promoter, secretion signals, protein tags, P4H variants) were subsequently ligated into multigene constructs (Additional File 1). We then used Cas12a-mediated transformation into the *A. niger* expression host Δ*gaaB-Mg*-17.2, an isolate which has been previously reported to produce the P4H cofactor ascorbic acid [21]. Homokaryotic transformants with correct targeted genomic integration of the respective expression cassette at the native *pyrG* locus (An12g03570) were verified via diagnostic PCR (Additional File 4) and are detailed in Table 1.

### Luciferase assay and fluorescent microscopy confirm collagen III expression in *A. niger*

**Fig. 2:**
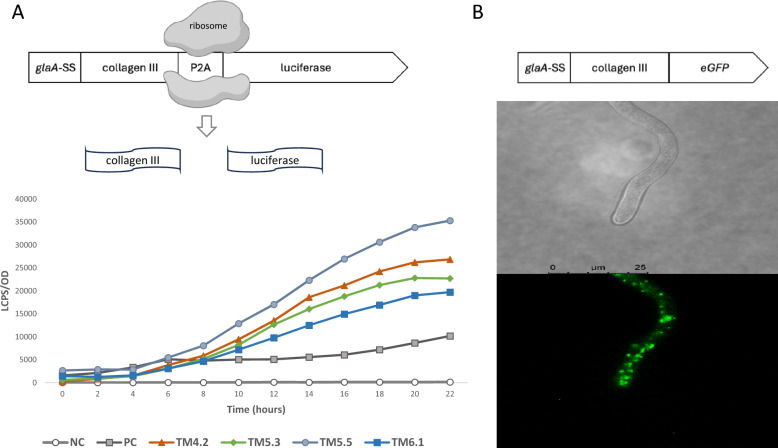
**A Luciferase luminescence as a reporter for collagen expression:** The P2A sequence between the collagen constructs and luciferase reporter gene causes ribosomal skipping during gene translation, resulting in no peptide bond formation between the glycine and proline residues within the P2A region. This produces equimolar translation of each peptide, collagen III and luciferase, under the Tet-on promoter. Strains containing Tet-on inducible collagen cassette were inoculated (5.0x10^6^ spores/mL) to a 96 well plate into CM (200μl) containing beetle luciferin (0.4 mM) and doxycycline (20 μg/mL). Luminescence was detected over 24 h using a Perkin Elmer 2030 Multilabel Reader VictorTM X3 with the average of experimental triplicates normalised to OD at 595 nm depicted. Negative control (NC) was uninoculated media, positive control (PC) was VG8.27 expressing luciferase under the Tet-on promoter. Reduction in luciferase expression at ~20 h was consistent with sporulation of these isolates causing subsequent blocking of luciferase detection. **B Fluorescence microscopy confirms confirms translation of eGFP tagged collagen III:** TM17.1 expressing a collagen III::eGFP cassette. Spores (1.0x10^6^) were cultivated for 24 h at room temperature on solid minimal medium containing 20 μg/mL doxycycline and fluorescence was measured with excitation at 450-490 nm and emission at 500-550 nm with a differential interference microscope (Leica)

In order to verify the functionality of the expression cassettes and to rule out unwanted gene silencing, we confirmed collagen transcription using a luciferase assay. Polycistronic cassettes (Fig. 1, Table 1) encoded 5′–3′: (i) the collagen gene; (ii) P2A sequence for ribosomal skipping, and (iii) a luciferase reporter (Fig. 1). Thus, we used detection of luciferase as a proxy measurement for collagen III transcription (Fig. 2A). Isolates TM4.2, TM5.3, TM5.5 and TM6.1 expressing collagen-P2A-luciferase and respective P4H variants (Table 1) displayed luminescence values well above those from the control isolate VG8.27 expressing luciferase under the Tet-on promoter. Next, we confirmed translation of the collagen products by generating isolate TM17.1 (Table 1) which expressed collagen III fused to a 3’ eGFP moiety. This allowed us to follow the intracellular fate of the recombinant collagen and assess protein localization. Fluorescence microscopy confirmed robust collagen translation, with the eGFP reporter suggesting highly abundant protein localized in punctate structures that accumulate at hyphal tips and are reminiscent of post-Golgi vesicles (Fig. 2B). Taken together, we conclude that collagen is efficiently expressed and translated in *A. niger*.

### Shake-flask fermentation using collagen expression isolate TM4.2 results in the presence of Aspergillopepsin A in culture supernatants

We initially tested collagen expression levels using isolate TM4.2, expressing collagen under the Tet-on promoter, and progenitor control *gaaB-Mg*-17.2 (Table 1) in simple shake-flask assays. Expression of collagen was induced with doxycycline (20 µg/mL) and media included D-galacturonic acid (GalUA, 20 g L^−1^). GalUA is hypothesized to be necessary for the P4H cofactor production in the progenitor isolate [21]. Total supernatant protein after 48 h cultivation were used in SDS-PAGE experiments to visualize proteins. A strong band from isolate TM4.2 was observed at approximately 45 kDa that was clearly absent from the progenitor control (Additional File 5). This was subsequently identified by LC–MS as Aspergillopepsin A (*pepA*, An14g04710). Thus, we hypothesized that extracellular proteolytic activity may be upregulated in the collagen expressing isolates leading to subsequent degradation of the recombinant collagen in the filtrates.

In order to reduce *pepA* expression, we generated isolate FGE2.1 with a point mutation in the *prtT* gene which is required for expression of multiple protease encoding genes in *A. niger* [36]. FGE2.1 was confirmed to be protease deficient by phenotypic screening on casein containing solid agar (data not shown). No growth defects were observed in the *prtT* mutant where non-proteinogenic nitrogen sources were present. Collagen expression cassettes encoding a collagen III/human P4H were then expressed in this background yielding isolate TM4.2.1 (Table 1).

### Hydroxylated collagen is detectable in the supernatant of an *A. niger* protease-deficient mutant

**Fig. 3:**
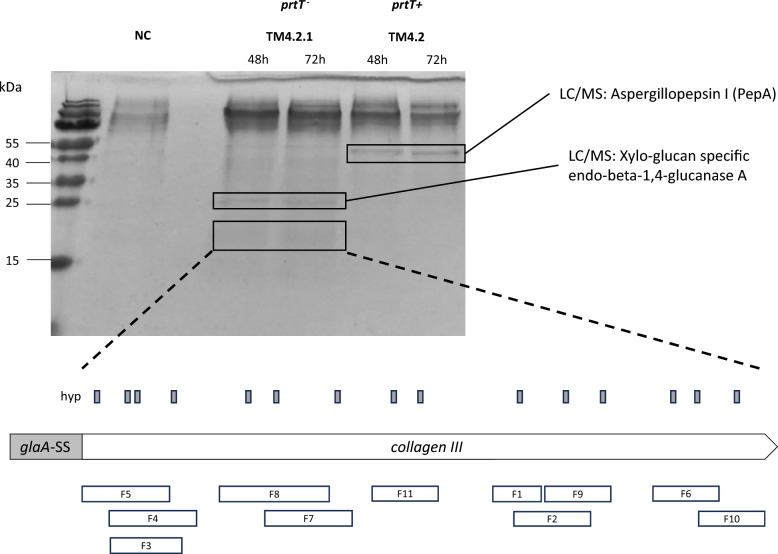
**Hydroxylated collagen III is detectable in the supernatant of the** ***prtT ***^***−***^ **mutant TM4.2.1:** Total proteins from culture filtrate (20 µl 5 × concentrated) from GalUA medium shake flask cultures inoculated with multiple collagen expressing strains were separated using SDS-PAGE (12%). Highlighted bands were excised and analysed by LC/MS for identification. Peptide fragments (F1–F11) from the 17-kDa band spanning the expected collagen III amino acid sequence were detected. The fragments indicated that a number of proline residues were hydroxylated (hyp, grey square) in the Y position of the Gly–X–Y tripartite repeat

Protease deficient collagen expressing isolate TM4.2.1 was cultivated in GalUA medium for 72 h for hydroxylated collagen production. Isolate TM4.2, expressing collagen with an unmutated *prtT* locus, and progenitor FGE2.1 were also evaluated (Table 1). Total proteins sampled from culture filtrates at 48 and 72 h post inoculation were assessed by SDS-PAGE (Fig. 3). As expected, *prtT* inactivation in isolate TM4.2.1 resulted in undetectable levels of PepA in the culture filtrate, which was clearly visible in *prtT* control isolates (Fig. 3).

A protein band with an approximate MW of 17 kDa was exclusively observed in the *prtT*^−^ collagen expressing isolate TM4.2.1. We hypothesized that this protein band could be recombinant collagen, which was excised and sent for identification by peptide sequencing. LC–MS analyses confirmed multiple collagenous peptide fragments (Additional File 6) as outlined in Fig. 3.

Additionally, multiple proline residues in the Y-position of the Gly-X–Y tripartite sequence of the collagenous fragments were shown to be hydroxylated (Fig. 3, Additional File 6). However, poor reproducibility between technical replicates and low protein titers required further optimization to increase collagen culture filtrate titers. Media optimization experiments to further reduce the extracellular degradation of collagen were thus performed.

### Media nitrogen source optimization reduces extracellular degradation of recombinant collagen

**Fig. 4:**
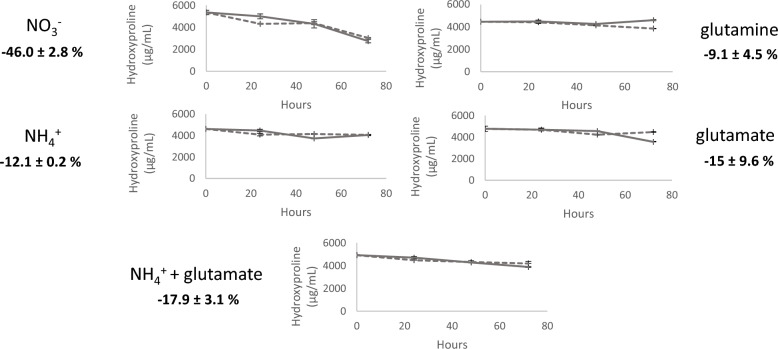
**Preferred nitrogen sources decrease supernatant degradation of hydrolysed collagen:** Supernatant hydroxyproline concentration as an indicator of the utilisation of collagen as a nitrogen source. Progenitor isolate FGE2.1 was inoculated in 50 mL shake-flask cultures containing 70 mM of various nitrogen sources and 1.5 mgL^−1^ Peptan^®^ (hydrolysed collagen, Rousselot, Belgium). Culture filtrate was sampled every 24 h up to 72 h. Percentage loss of collagen calculated as the average loss in hydroxyproline relative to concentration at t=0 of biological and experimental duplicates.

Carbon and nitrogen sources within cultivation media have large effects on gene transcription in *Aspergilli* [37] primarily due to the repressive mode of action of carbon catabolite repression (CCR) and nitrogen catabolite repression (NCR). Similarly, NCR has recently been utilized to control gene expression in *A. nidulans* [38]. *A. niger* is conventionally grown in non-repressing nitrogen sources, i.e., nitrate. We postulated change of growth media to a repressing nitrogen source would induce transcriptional repression of catabolic genes involved in nitrogen assimilation (i.e., proteases), thus alleviating degradation of proline-rich collagen. In order to test this hypothesis, isolate FGE2.1 was cultivated in shake flasks containing 1.5 mgL^−1^ Peptan^®^ (i.e., hydrolysed collagen) and 70 mM of various nitrogen sources (Fig. 4).

*A. niger* breakdown and uptake of the spiked collagen was assessed by quantifying the average reduction in media hydroxyproline content from biological and experimental duplicates after 72 h. These results confirmed supplementation of ammonium to cultivation media successfully reduced collagen degradation approximately fourfold (12% loss vs 46% in NO_3_^−^, Fig. 4). This was consistent with previously reported data in *A. niger* [39], where ammonium use also resulted in lower extracellular protease activity than glutamine. Correspondingly, all further cultivation media were supplemented with ammonium as the sole nitrogen source, consistent with bioreactor medium cultivations [40].

### Reconfiguring expression systems for constitutive HiBiT tagged collagen III production

**Fig. 5:**
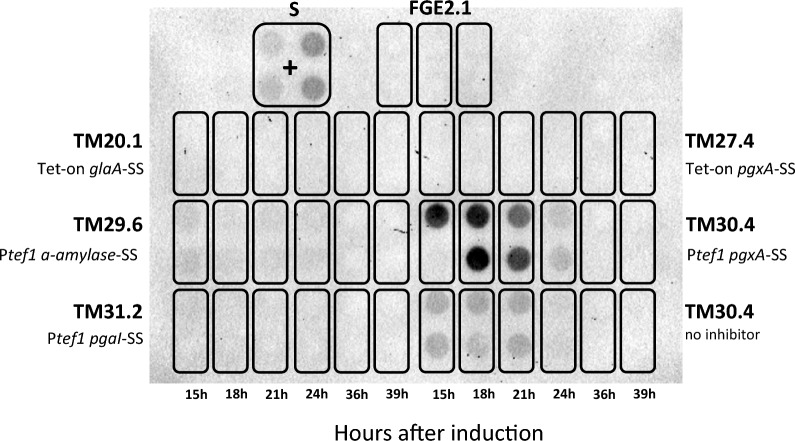
***A. niger*** **strains expressing collagen III under the** ***tef1*** **promoter secrete the protein into the supernatant A. Dot Blot HiBiT Assay:** Isolates inoculated to 50 mL CM (1% Glucose) and grown for 96 hours at 30°C and 200 rpm with protease inhibitors (Pierce, Roche). 100 μl supernatant was mixed 1:1 with 1xPBS and 200 μl was added to the 0.2 μM nitrocellulose membrane via vacuum filtration before use in the HiBiT detection assay (Promega). Membrane was imaged using the ChemiDoc imager (BioRad) with cumulative image acquirement, 30 s intervals for 5 min. S – supernatant; P – promoter; SS – secretion signal

Although the Tet-on promoter was successfully used for initial proof-of-principle experiments, this approach is not suitable for scale-up or industrial applications as antibiotic addition to growth media is costly and can co-purify with the desired product. Consequently, further optimization of the expression system using native promoters and secretion signals was performed. Additionally, given that protein visualization using SDS-PAGE indicated that recombinant collagen supernatant titers were low (Fig. 3), we decided to utilize the HiBiT^®^ tag (Promega) for reproducible and accurate detection. HiBiT^®^ is an 11-amino acid epitope capable of producing bioluminescence when bound to its complementation partner LgBiT, which has previously been validated for detection at a picomolar scale [41]. The HiBiT^®^ epitope tag was fused at the C-terminus of collagen III to avoid complications with the N-terminal secretion signal (Table 1). Expansion of the plasmid library was thus implemented to encode a constitutive promoter belonging to the translation elongation factor 1 (*tef1*, An18g04840) and various N-terminal secretion signals (Table 1). The alpha amylase secretion signal has been shown to be stronger than the *glaA* secretion signal [42]. Growth in pectin or D-GalUA results in the induction of pectinases [43], and it was postulated that these may be preferentially secreted. Thus, the secretion signals from the most highly expressed pectinases in *A. niger* were also tested. Numerous combinations of the promoters/secretion signals were cloned and expressed in *A. niger*, with shake-flask assays of collagen production revealing isolate TM30.4 had the strongest supernatant signal of HiBiT^®^ tagged collagen III (Fig. 5). This isolate expressed collagen using the *tef1* promoter and a *pgxA* (An11g04040) secretion signal. We thus used this isolate for further optimization.

### Comparative transcriptomic analyses show increased expression of the endopeptidase gene *protA* during collagen expression

**Fig. 6:**
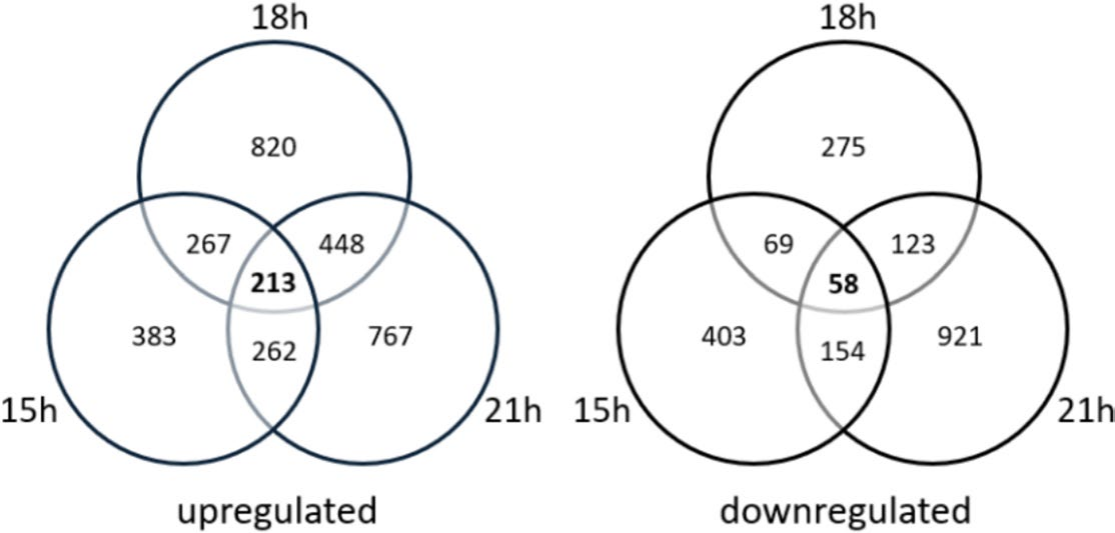
**A proline specific endopeptidase,** ***protA,*** **is highly upregulated in the collagen-secreting isolate TM30.4.** Comparative transcriptomic analyses for differential gene expression between TM30.4 and the protease deficient progenitor FGE2.1 were performed. Isolates were cultivated in 50 mL CM shake flask and total RNA samples extracted at t = 15, 18 and 21 h after inoculation, based on previous supernatant signal observed (Supplementary Fig. 1). High stringency differential gene expression was performed with a mean raw count > 200, a log_2_ fold-change > 1 (upregulated) or log_2_ fold-change < − 1 (downregulated) and a multiple hypothesis corrected p-value < 0.05

We hypothesized that protease activity was still limiting titers of recombinant collagen. Collagen expressing isolate TM30.4 and progenitor control FGE2.1 were thus cultivated in shake flasks containing complete medium (CM) for comparative transcriptomic analyses. Based on previous extracellular collagen-HiBiT signal stability (Fig. 5), total RNA was extracted and sequenced using Illumina sequencing (see Materials and methods) at *t* = 15, 18 and 21 h. Differentially expressed genes were defined based on fold change (log_2_ fold-change > 1.0 or < − 1.0) which identified 213 genes upregulated and 58 downregulated in the collagen-secreting isolate TM30.4 compared to the progenitor FGE2.1 at all three time points (Fig. 6). A selection of Gene Ontology (GO) Terms from the sampling point corresponding to the strongest collagen signal is presented in Table 2.

**Table 2:** Selection of Gene Ontology (GO) terms

GO term	GO ID	GO term description	Number of genes	Fold enrichment		Genes in GO Term		p-value
BP	GO:0006950	Response to stress	105		1.5		615	9.47e−6
BP	GO:0006984	ER-nucleus signalling pathway	5		4.41		10	2.99e−3
BP	GO:0006810	Transport	179		1.19		1323	5.60e−3
BP	GO:0071692	Protein localization to extracellular region	6		3.31		16	6.18e−3
BP	GO:0006508	Proteolysis	40		1.36		259	2.64e−2
CC	GO:0016021	Integral component of membrane	287		1.19		2094	4.99e−4
CC	GO:0000323	Lytic vacuole	28		1.78		139	1.73e−3

Biological processes (BP) or cell component (CC) upregulated from a low-stringency analysis in the collagen-secreting strain TM30.4 relative to the expression host FGE2.1 at 18 h after cultivation

Overall, the data suggest that production of collagen results in increased cellular stress, while also increasing genes involved in protein localization to the extracellular region and a large number of genes related to transport and proteolysis (see Additional File 7 for complete gene list). Upregulation of genes involved in the lytic vacuole hint at potential intracellular degradation or recycling of recombinant collagen. Interestingly, none of the key genes involved in the Unfolded Protein Response (UPR) [44] or Endoplasmic Reticulum Associated Degradation (ERAD) [45] appeared to be upregulated. Most strikingly, a secreted lysosomal Pro-Xaa endopeptidase, *protA*, was found to be significantly upregulated in the collagen-secreting strain by a log_2_ fold-change of 2.00, 1.43 and 1.46 at 15, 18 and 21 h after inoculation, respectively. Given the high proline content of the expressed collagen construct, we assumed this protease was responsible for the apparent extracellular degradation observed (Fig. 5). Thus, *protA* (An08g04490) was deleted in isolate FGE2.1 using two Cas12a directed protospacers to yield the protease deficient, *pyrG*^−^, Δ*protA* strain AT2.1.

### A ∆*protA* mutant secretes partially hydroxylated collagen with increased supernatant stability

**Fig. 7:**
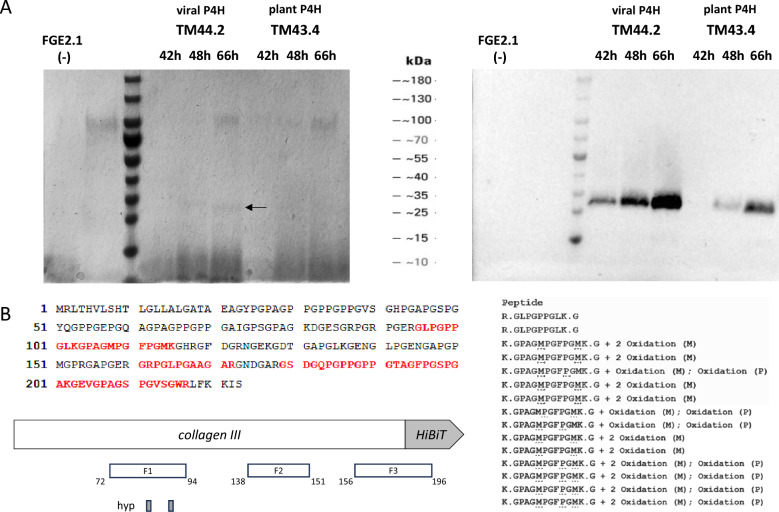
**∆*protA*** **isolate TM44.2 expressing a viral P4H secretes partially hydroxylated collagen with increased supernatant stability.** **A** Total unmodified culture filtrate proteins (15 μL) from shake-flask cultures of GalUA medium supplemented with 5% glucose were separated by SDS-PAGE (4-15%, Biorad) and either transferred to nitrocellulose for HiBiT blotting (right) or stained for 30 min in Coomassie G in 30 mM HCl (left). Arrows indicate bands extracted for analysis by LC-MS. **B** Lower protein band marked in A was excised and sent for protein identification using LC-MS against the MASCOT database and A. niger secretome, allowing for mass changes corresponding to hydroxylation of proline residues. The peptide fragments (F1-F3) identified are marked in red in the expected amino acid sequence of the HiBiT tagged collagen III. Oxidation (hydroxylation) present on methionine (M) or proline (P) residues is underlined (right). Hydroxylated proline residues (hyp) marked in grey (left)

Transforming the Δ*protA* progenitor with the collagen cassettes (P*tef1*-*pgxA*-SS-collagen III-HiBiT) expressing plant or viral P4Hs yielded two new isolates, TM43.4 and TM44.2, respectively (Table 1). These isolates were assessed for collagen production in the optimized GalUA medium (NH_4_^+^) supplemented with 5% glucose (Fig. 7). Collagen III-HiBiT signal was detected and observed to stably increase over time from culture filtrates of both strains at an approximate molecular weight (MW) of 30 kDa (Fig. 7A). It was notable that expression of the viral-derived PH4 in isolate TM44.2 resulted in higher titers of collagen III. It was clear that the molecular mass of approximately 30 kDa is much larger than expected for collagen III (19 kDa). While hydroxylation of the proline residues in collagen is only expected to result in a small increase molecular mass, it has been shown to hinder migration of the protein in SDS-PAGE [46]. Thus, this increase in apparent MW suggested hydroxylation of the recombinant protein. The protein band corresponding to the strongest collagen-HiBiT signal observed from isolate TM44.2 (66 h, Fig. 7A) was analysed by LC–MS, assessing hydroxylation (oxidation) of the amino acid residues present in the detected fragments. Peptide fragments covering 38% of the expected amino acid sequence of expressed collagen III were identified (Fig. 7B, left). Partial, but not total, hydroxylation of proline residues in the Y position of the Gly-X-Y tripartite was additionally observed (Fig. 7B, right), accounting for 16% of the expected proline residues to be hydroxylated. Additional File 8 highlights the HiBiT-Western Blot that was used to semi quantitatively calculate the concentration of secreted collagen III by TM44.2 to be 5 mgL^−1^ using ImageJ. This showed stable expression in the *ΔprotA* isolate compared to the *protA* WT for up to 140h (Additional File 9).

Masses corresponding to both hydroxylated and non-hydroxylated forms of these protein fragments highlighted heterogeneity within the recombinant protein. These results support the hypothesis that deletion of *protA* was successful in increasing supernatant stability of the secreted collagen III. Engineered isolates reproducibly showed stably increasing, detectable levels of supernatant HiBiT signal (see loss of signal after 24 h, Fig. 5), highlighting the effectiveness of these genetic optimizations. Additional File 9 shows stable signal increase up to 140 h. Accordingly, protein titers were now reproducibly increased, allowing LC–MS confirmation that partial hydroxylation occurs in the isolate TM44.2.

## Discussion

The current standard of industrial collagen production from animal sources no longer aligns with the modern-day requirements of a virus free, renewable collagen source. Microbial sources of collagenous proteins, contrastingly, meet these necessary criteria, offering controllable, virus-free protein production from growth on renewable crops or agricultural byproducts. Thus, recombinant collagen from *A. niger* can constitute an excellent option for use in wound healing, tissue printing and cosmetics. This study has proven for the first time that *A. niger* is capable of producing recombinant and hydroxylated collagen and the steps necessary to achieve this (Additional File 10).

### Modular cloning facilitated high-throughput construction of a collagen library

With regard to the methodological basis of this study, the modular cloning strategy undertaken was instrumental in providing high-throughput assembly of multigene constructs. Similar approaches have been demonstrated in other industrially used fungal systems [47]. It allowed the construction of a suite of collagen expressing cassettes conveying a range of modifications to promoter, secretion signal, polycistronic constructs and protein tags within a single reaction tube. Similarly, future strain generation will benefit greatly from the already existing library.

It should be noted that the collagen III transcript chosen was a truncated form of COL3A from *Sus scrofa*. This approach was utilized to increase the chances that the recombinant molecule could physically fit within COPII vesicles during classical secretion [48]. While the COL3A1 fragment does contain two GXOGER motifs (see Additional File 3) for interactions with cell membranes [49], there may be further functional motifs encoded in the native full-length protein that increase effectiveness in wound healing or tissue printing applications. The full COL3A1 sequence will be tested in future studies.

### Molecular evidence confirmed collagen production

Isolates expressing the polycistronic collagen III-P2A-luciferase construct were used to confirm translation of collagen (Fig. 2A). While constructs are expected to produce equimolar mRNA, being encoded by a single transcript, variations in luciferase activity corresponding to the position in the construct have been reported [34]. Thus, the luciferase assay could provide evidence of collagen translation, but was unsuitable for quantification of collagen. Supporting this, it has also been shown that N-terminal proline present after ribosomal skipping at the P2A sequence affects protein stability and stoichiometry [50]. Isolates expressing an eGFP-tagged collagen III provided further evidence of intracellular presence of the recombinant protein. While possibly being enriched in the post-Golgi cisternae (Fig. 2), fluorescence microscopy evidence could not support concentration at the Spitzenkörper in the hyphal tip (Fig. 2B). Given the upregulation of genes involved in lytic vacuoles in collagen expressing strains (Additional File 7), these experiments could hint at the possibility that collagen III is subsequently recycled intracellularly. Further co-localization experiments such as those presented by Fiedler et al. [51] would be required to follow the exact intracellular fate of the recombinant collagen III.

### Genetic and media optimizations improved collagen transcription and stability

We have shown here three successful engineering and optimization approaches to improve collagen supernatant titers and stability.

Firstly, engineering of the progenitor host to protease deficiency (*prtT*^−^) provided evidence of reduced extracellular proteases, including loss of Aspergillopepsin A secretion, resulting in a faint band at the expected MW of the procollagen expressed (Fig. 3). Furthermore, deletion of the proline specific endopeptidase, *protA,* resulted in reproducibly increasing supernatant titers of collagen III up to 66 h (Fig. 7). Protease inhibitors were not effective at stopping the extracellular degradation in previous isolates (see Fig. 5), consistent with literature findings which is why this upregulated protease was chosen for deletion [52]

Secondly, we provide evidence that utilizing intrinsic nitrogen catabolite repression with the addition of a repressing nitrogen source to the growth medium successfully reduced collagen degradation (Fig. 4), presumably through repression of genes involved in nitrogen assimilation [38].

Thirdly, we optimized the collagen expression system through expansion of the collagen expression library to include native, constitutive promoter *tef1* and multiple secretion signals paired with a sensitive HiBiT^®^ epitope tag to overcome issues of low expression and collagen detection at low titers. The HiBiT^®^ tag had not been previously, until recently [53], established for use in filamentous fungi. This library expansion provided the isolate TM30.4, producing the strongest observed supernatant filtrate collagen III-HiBiT signal under the *tef1* promoter fused to the *pgxA* secretion signal.

### Effects of collagen production on the transcriptional landscape of *A. niger*

qPCR experiments were used to confirm collagen transcription in multiple isolates (see Additional File 11). Differential gene expression analysis between TM30.4 and the progenitor FGE2.1 provided substantial evidence that constitutive collagen expression provokes intracellular stress to *A. niger*. Interestingly, investigation of expression levels of the major genes involved in ERAD and the UPR all appear to be significantly downregulated (Additional File 7). Protein disulfide isomerases are an integral part of the UPR machinery [45], catalysing disulfide bond formation between cysteine residues to increase protein folding [54]. However, overexpression of these enzymes is not expected to function on collagen due to the lack of cysteine residues, which may explain the downregulation observed. ERAD components remove misfolded proteins from the ER and target them for degradation [45]. There is also evidence in *A. niger* that glucose deplete conditions leads to re-targeting of proteins from the secretory pathway to vacuoles for recycling [55]. Pairing transcriptional evidence in this study of upregulated lytic vacuoles and cytoplasm-to-vacuole targeting (CVT), we hypothesize that ERAD components are downregulated, and intracellular collagen may be targeted to vacuoles for recycling rather than degradation by the proteasome. Comparative transcriptomics also revealed a protease, *protA,* was strongly upregulated at all time points in the collagen expressing strain compared to the progenitor (Fig. 6). Evidence that deletion of this protease increased supernatant stability of the expressed collagen III is presented here (Fig. 7, Additional Files 8 and 9). Isolates with an unmodified *protA* (Fig. 5) showed loss of supernatant collagen III-HiBiT signal between 24 and 36 h, whereas Δ*protA* isolates TM44.2 and TM43.4 displayed stable, increasing supernatant signal up to 66 h (Fig. 7). The isolate TM39.8 expressed a human P4H and illustrated supernatant stability up to 140 h (Additional File 9). While quantitative comparisons cannot be made between the blots, TM44.2 was shown to qualitatively produce a distinctly larger protein band (Fig. 7) estimated at 5 mg L^−1^.

### Isolate TM44.2 secreted partially hydroxylated recombinant collagen

Assessment of the culture filtrate proteins from isolate TM44.2 (Δ*protA* collagen secretor, viral P4H) indicated partial hydroxylation of the proline residues in the Y position of the Gly-X–Y tripartite repeat, amounting to 16% of the expected hydroxylation pattern. No other PTMs were assessed with this method. While lysine hydroxylation could be expected for collagen [56], no known lysine hydroxylase has been identified from *A. niger*. LC–MS cannot distinguish between hydroxylation of the methionine and proline residues. Though the degree of proline hydroxylation could therefore be interpreted as higher, we present the minimum value here. While hydroxylation of 40% of proline residues is expected in eukaryotes [57], the partial hydroxylation observed was well below this. Heterogeneity of hydroxylation was also observed, but has also been reported in eukaryotes [58]. Firstly, 42% of the amino acid sequence from the queried protein (Fig. 7B) was not detected, which encodes a higher proportion of proline residues expected to be hydroxylated. Secondly, we propose that genetic modifications performed to the galacturonic acid transporter *gatA* in the isolate TM44.2 allowing transcription in glucose replete conditions may be partly responsible. Preliminary evidence (HPLC, data not shown) suggested that D-galacturonic acid (D-GalUA) may be depleted after 24 h of cultivation. Given the role of D-GalUA in restoring P4H enzymatic competence, additional medium optimization for these shake-flask experiments needs to be investigated to overcome these shortcomings. We suggest adjusting process parameters regarding D-GalUA availability could be successful in restoring P4H activity and thus hydroxylation of the recombinant collagen. Further transcriptomic analyses are currently underway to uncover targets to reduce the intracellular stress found to be upregulated in the GO Terms (Table 2). Additionally, these will be used to assess potential targets for genetic modifications aiming to increase collagen titre as well as hydroxylation levels.

## Conclusion

This study has shown that the multipurpose cell factory *A. niger* can be engineered to secrete partially hydroxylated recombinant collagen. The genetic engineering and medium optimization experiments described in this study were successful at increasing titre and extracellular stability of the recombinant collagen. The strains and approaches we have developed constitute a vital step towards generating high titres of virus-free collagen in the future. Additional comparative transcriptomic analyses aim to detect further targets for genetic modification and increase collagen titre to those that are viable for large scale production. Likewise, we predict that future process parameter optimization will increase hydroxylation levels of the recombinant collagen to that of the native protein.

## Supplementary Information

Additional file 1. Plasmids used in this study.

Additional file 2. Primers used in this study.

Additional file 3. Recombinant Protein sequences.

Additional file 4. PCR proofs of cassette integration at the target locus.

Additional file 5. SDS-PAGE of total proteins from *A. niger* isolates expressing collagen.

Additional file 6. Collagenous fragments obtained from LC-MS data of extracted 17 kDa band from *A. niger* isolate TM4.2.1.

Additional file 7. Transcriptomic tables.

Additional file 8. *A. niger* strains expressing collagen III under the *tef1* promoter secrete the protein into the supernatant.

Additional file 9. *A. niger* strains secrete HiBiT tagged collagen III with supernatant stability up to 140 h.

Additional file 10. Schematic of genetic engineering steps to construct the recombinant collagen secreting isolate *A. niger* strain TM44.2 from the progenitor strain ∆*gaaB*.

Additional file 11. qPCR results from strains expressing collagen III.

## Data Availability

All data generated or analysed during this study are included in this manuscript and its additional information files. All raw sequencing data are available at NCBI SRA database under PRJNA1229437.
