# Supplementary material for: The multipurpose cell factory Aspergillus niger can be engineered to produce hydroxylated collagen
Source: Biotechnol Biofuels Bioprod. 2025 Aug 8;18:88. doi: 10.1186/s13068-025-02681-y (PMC12333218; doi:10.1186/s13068-025-02681-y)
Supplement: Supplementary file 1 — Additional file 1. Plasmids used in this study. [file 13068_2025_2681_MOESM1_ESM.docx]

P – promoter, T- terminator.

**Level 0**

| Name | description | backbone |  |
| --- | --- | --- | --- |
| pTM_0_2x | *pdiA*SS-human*P4H*alpha-P2A-*bipA*SS-human*P4H*beta | plCH41308 |  |
| pTM_0_3x | plant-*P4H* | plCH41308 |  |
| pTM_0_4x | viral-*P4H* | plCH41308 |  |
| pTM_0_5 | *glaA*SS-colIII-P2A-*luc* | plCH41308 |  |
| pTM_0_10 | *glaA*SS-colIII:*eGFP* | plCH41308 |  |
| pTM_0_11 | *glaA*SS-colIII:HiBiT | plCH41308 |  |
| pTM_0_14 | α-amylaseSS::colIII::HiBiT | plCH41308 |  |
| pTM_0_15 | *pgxA*SS::colIII::HiBiT | plCH41308 |  |
| pTM_0_16 | *pgaI*::colIII::HiBiT | plCH41308 |  |
|  |  |  |  |
| Level 1 |  |  |  |
| Name | **description** | **backbone** |  |
| pVN_1_3 | P*gpdA-rtTA2-MS*-T*cgrA* | plCH47751 |  |
| pTM_1_2x | P*tef1*-human*P4H*alpha-P2A-human*P4H*beta-T*amdS* | plCH47761 |  |
| pTM_1_3x | P*tef1*-plant*P4H*-T*amdS* | plCH47761 |  |
| pTM_1_4x | P*tef1*-viral*P4H*-T*amdS* | plCH47761 |  |
| pTM_1_5 | Tet-on-colIII-P2A-*luc*-T*trpC* | plCH47732 |  |
| pTM_1_7 | P*pyrG*-*AopyrG*-T*cpY* | plCH47742 |  |
| pTM_1_8 | Tet-on-colIII::*eGFP*-T*trpC* | plCH47732 |  |
| pTM_1_15 | Tet-on-colIII::HiBiT-T*trpC* | plCH47732 |  |
| pTM_1_16 | Tet-on-alphaamylaseSS::colIII::HiBiT-T*trpC* | plCH47732 |  |
| pTM_1_17 | Tet-on-*pgxA*SS::colIII::HiBiT-T*trpC* | plCH47732 |  |
| pTM_1_18 | Tet-on-*pgaI*SS::colIII::HiBiT-T*trpC* | plCH47732 |  |
| pTM_1_19 | P*tef1*-alphaamylaseSS::colIII::HiBiT-T*trpC* | plCH47732 |  |
| pTM_1_20 | P*tef1*-*pgxA*SS::colIII::HiBiT-T*trpC* | plCH47732 |  |
| pTM_1_21 | P*tef1*-*pgaI*SS::colIII::HiBiT-T*trpC* | plCH47732 |  |
| Level 2 |  |  |  |
| Name | **description** | | |
| pTM_2_1 | GPPG6-P2A-*luc*-*AopyrG*-*rtTA2S-M2*-human*P4H*alpha-P2A-human*P4H*beta | | |
| pTM_2_2 | GPPG6-P2A-*luc*-*AopyrG*-*rtTA2S-M2*-plant*P4H* | | |
| pTM_2_3 | GPPG6-P2A-*luc*-*AopyrG*-*rtTA2S-M2*-viral*P4H* | | |
| pTM_2_4 | colIII-P2A-*luc*-*AopyrG*-*rtTA2S-M2*-human*P4H*alpha-P2A-human*P4H*beta | | |
| pTM_2_5 | colIII-P2A-*luc*-*AopyrG*-*rtTA2S-M2*-plant*P4H* | | |
| pTM_2_6 | colIII-P2A-*luc*-*AopyrG*-*rtTA2S-M2*-viral*P4H* | | |
| pTM_2_11 | colIII-*AopyrG*-*rtTA2S-M2*-TU4dummy | | |
| pTM_2_13 | colIII::*eGFP*-*AopyrG*-*rtTA2S-M2*-human*P4H*alpha-P2A-human*P4H*beta | | |
| pTM_2_14 | colIII::*eGFP*-*AopyrG*-*rtTA2S-M2*-plant*P4H* | | |
| pTM_2_15 | colIII::*eGFP*-*AopyrG*-*rtTA2S-M2*-viral*P4H* | | |
| pTM_2_16 | colIII::HiBiT-*AopyrG*-*rtTA2S-M2*-human*P4H*alpha-P2A-human*P4H*beta-LgBiT | | |
| pTM_2_17 | colIII::HiBiT-*AopyrG*-*rtTA2S-M2*-human*P4H*alpha-P2A-human*P4H*beta | | |
| pTM_2_18 | Tet-on-alphaamylaseSS::colIII::HiBiT-*AopyrG*-*rtTA2S-M2*-human*P4H*alpha-P2A-human*P4H*beta | | |
| pTM_2_19 | Tet-on-*pgxA*SS::colIII::HiBiT-*AopyrG*-*rtTA2S-M2*-human*P4H*alpha-P2A-human*P4H*beta | | |
| pTM_2_20 | Tet-on-*pgaI*SS::colIII::HiBiT-*AopyrG*-*rtTA2S-M2*-human*P4H*alpha-P2A-human*P4H*beta | | |
| pTM_2_21 | P*tef1*-alphaamylaseSS::colIII::HiBiT-*AopyrG*-*rtTA2S-M2*-human*P4H*alpha-P2A-human*P4H*beta | | |
| pTM_2_22 | P*tef1*-*pgxA*SS::colIII::HiBiT-*AopyrG*-*rtTA2S-M2*-human*P4H*alpha-P2A-human*P4H*beta | | |
| pTM_2_23 | P*tef1*-*pgaI*SS::colIII::HiBiT-*AopyrG*-*rtTA2S-M2*-human*P4H*alpha-P2A-human*P4H*beta | | |
| pTM_2_24 | P*tef1*-*pgxA*SS::colIII::HiBiT-*AopyrG*-*rtTA2S-M2*-plantP4H | | |
| pTM_2_25 | P*tef1*-*pgxA*SS::colIII::HiBiT-*AopyrG*-*rtTA2S-M2*-viralP4H | | |
| pTM_2_26 | P*httA-DR-AopyrG-DR* | | |
| pTM_2_27 | P*rpl15*- *DR-AopyrG-DR* | | |

**Plasmids used within this study**ampR - confers resistance against ampicillin

| name | description | MoClo backbone |
| --- | --- | --- |
| pMC_0_78 | P*gpdA*_core | plCH41295 |
| pMC_0_12 | *rtTA2-MS* | plCH41308 |
| pMC_0_15 | T*cgrA* | plCH41276 |
| pMC_0_10 | T*trpC* | plCH41276 |
| pMC_0_13 | Ptet-7x | plCH41295 |
| pMC_0_62 | TcpyY | plCH41276 |
| pMC_0_69 | Firefly-*luc* | plCH41308 |
| pMC_0_76 | PpyrA | plCH41295 |
| pMC_0_77 | *pyrG* | plCH41308 |
| pMC_1_74 | P*pyrG*-*AopyrG*-Thxk | plCH47742 |
| pLB5.1 | a*mpR* backbone | - |
| pMC_1_135 | Ptet6x-LgBiT-T*ade1* | plCH47772 |
| plCH41766 | TU-3 end linker | - |
| plCH41780 | TU-4 end linker | - |
| plCH41800 | TU-5 end linker | - |
| plCH54044 | TU-4 dummy | - |
| pMC_0_14 | *eGFP* | plCH41308 |
| pMC_0_15 | T*amdS* | plCH41276 |
| pMC_0_46 | P*rpl15* | plCH41295 |
| pMC_0_50 | ThxK | plCH41276 |
| pMC_0_68 | P*tef1* | plCH41295 |
| pMC_0_74 | *eGFP*-SKL | plCH41308 |
| pMC_0_112 | *HsHSP47* | plCH41308 |
| pAK_1_4 | P*rpl15*-*HsHSP47*-T*hxk* | plCH47751 |
| pMC_1_67 | T*AopyrG*-as-DR | plCH47732 |
| pMC_1_66 | T*AopyrG*-as-DR | plCH47751 |
| pMC_1_77 | T*AopyrG*-as-DR | plCH47761 |
| pMC_1_76 | T*AopyrG*-as-DR | plCH47742 |
| pMC_1_103 | DR-*AopyrG*-DR | plCH47761 |
| pMC_1_106 | DR-*AopyrG*-DR | plCH47761 |
| pMC_0_13 | Tet-on-6x | plCH41295 |
