# Supplementary material for: The multipurpose cell factory Aspergillus niger can be engineered to produce hydroxylated collagen
Source: Biotechnol Biofuels Bioprod. 2025 Aug 8;18:88. doi: 10.1186/s13068-025-02681-y (PMC12333218; doi:10.1186/s13068-025-02681-y)
Supplement: Supplementary file 2 — Additional file 2. Primers used in this study. [file 13068_2025_2681_MOESM2_ESM.docx]

| \| Modular Cloning for expression plasmids \| \| \|  \| \| --- \| --- \| --- \| --- \| \| 2,774 \| human alpha P4H FW \| cggaagacaattctccctcctgaagcaggccggtgacgtcgaggagaaccccggccccATGGCGCGCATCTCGC \|  \| \| 2,775 \| human alpha P4H R \| cggaagacaacggagccCTCGAGCTCGCTCAGAGTACA \|  \| \| 2,776 \| human beta P4H FW \| cggaagacaatccggcgccaccaacttctccctcctgaagcaggccggtgacgtcgaggagaaccccggccccATGCGCTCCTTCGCGC \|  \| \| 2,777 \| human P4H beta unit R \| cggaagacaatcaggagggagaagttggtggcgccggagccAAGTTCGTCTTTTACAGCCTTCTGATCG \|  \| \| 2,778 \| luciferase FW \| cggaagacaactgaagcaggccggtgacgtcgaggagaaccccggcccccggaagacaactgaagcaggccggtgacgtcgaggagaaccccggccccATGGTCACCGACGCCA \|  \| \| 2,779 \| luciferase R \| cggaagacaaaagcCACGGCGATCTTTCCGCCC \|  \| \| 2,780 \| Plant PH4 R \| cggaagacaatcaggagggagaagttggtggcgccggagccTGACGTCGCCTTCTGGCG \|  \| \| 2,781 \| Viral P4H R \| cggaagacaatcaggagggagaagttggtggcgccggagccCTTCACTGCTCTGATCCATTGGTTGG \|  \| \| 2,782 \| collagen type III FW \| cggaagacaaaatgatgtcgttccgatctctactcgccctgagcggcctcgtctgcacagggttggcaGGTTATCCTGGTCCCGCCG \|  \| \| 2,783 \| collagen type III R \| cggaagacaaagaagttggtggcgccggagccTCCCGGGCTGCCAGC \|  \| \| 2,804 \| luciferase 4a FW \| gacgtcgaggagaaccccggccccATGGTCACCGACGCCA \|  \| \| 2,807 \| luciferase 4b FW \| cggaagacaactgaagcaggccggtGACGTCGAGGAGAACCCC \|  \| \| Screening primers \| \| \|  \| \| 2,824 \| lvl0alphascreenA \| GAAGGACGTGAACAAGAGTGC \|  \| \| 2,825 \| lvl0alphascreenB \| GCCAGGAGTTTCGTCGCC \|  \| \| 2,826 \| lvl0betascreen \| CACCGACAATCAACGCATAC \|  \| \| 2,827 \| lvl0lucifscreen \| CGGAGTTGCAGTTGCG \|  \| \| 2,828 \| lvl0plantscreen \| CCTAGCTGGTGACGGCG \|  \| \| 2,829 \| lvl0viralscreen \| GAGACTCTGCATGCTGGAC \|  \| \| Making ΔpyrG expression host \| \| \|  \| \| 2,830 \| An16g05390-5'-F \| TGGACGACTACTATGCCGAC \|  \| \| 2,831 \| An16g05390-5'-R \| gcattaagtggcggtaggtgCTGGGCTGGGGAAGTTACTT \|  \| \| 2,832 \| An16g05390-3'-F \| CACCTACCGCCACTTAATGC \|  \| \| 2,833 \| An16g05390-3'-R \| CAACACCGACAATGCTTCCA \|  \| \| 2,834 \| An16g05390-Con-F \| GCGTGCAGGTTCCTAAAGAC \|  \| \| 2,835 \| An16g05390-Con-R \| TGGTCGGATTGGGATTGGAA \|  \| \| 2,838 \| luciferase-4C-fw \| cggaagacaattctccctcctgaagcaggccggtGACGTCGAGGAGAACC \|  \| \| 2,839 \| alpha_x-fw \| cggaagacaaaatgATGGCGCGCATCTCGC \|  \| \| 2,840 \| beta_x-rev \| cggaagacaaaagCAAGTTCGTCTTTTACAGCCTTCTGATCG \|  \| \| 2,841 \| plant_x-fw \| cggaagacaaAATGATGGCGCGCATCTCGC \|  \| \| 2,842 \| plant_x-rev \| cggaagacaaaagCTGACGTCGCCTTCTGGCG \|  \| \| 2,843 \| viral_x-fw \| cggaagacaaaatgATGGCGCGCATCTCGC \|  \| \| 2,844 \| viral_x-rev \| cggaagacaaaagcGCTTCACTGCTCTGATCCATTGGTTGGC \|  \| \| 2,845 \| eGFP-a fw \| cggaagacaaTTCTCCCTCCTGAAGCAGGCCGGTGACGTCGAG \|  \| \| 2,846 \| eGFP - b fw \| ggccggtgacgtcgaggagaaccccggccccGTGAGCAAGGGCGAG \|  \| \| 2,857 \| P2A seq R \| AGAAGTTGGTGGCGCCGGAG \|  \| \| 2,858 \| PCR10-luc-R \| cggaagacaaATGGGGCCGGGGTTCTCCTC \|  \| \| 2,859 \| PCR10b-eGFP-R \| cggaagacaaacGGGGCCGGGGTTCTCCTC \|  \| \| 2,860 \| PCR11-luc-fw \| cggaagacaaccATGGTCACCGACGCCAAGAACATC \|  \| \| 2,861 \| eGFP-fw \| cggaagacaaccGTGAGCAAGGGCGAGGAGCTG \|  \| \| 2,862 \| PCR13-R \| cggaagacaaCCTCCCGGGCTGCCAGCAGG \|  \| \| 2,863 \| PCR14-colIIIback-R \| cggaagacaaAAGCTCCCGGGCTGCCAGCAG \|  \| \| 2,864 \| PCR15-P2AcolIII-fw \| cggaagacaagaGGCTCCGGCGCCACC \|  \| \| 2,865 \| PCR15-P2A-luc-R \| cggaagacaataGGGGCCGGGGTTCTCCTC \|  \| \| 2,866 \| PCR15-P2A-eGFP-R \| cggaagacaaacGGGGCCGGGGTTCTCCTC \|  \| \| 2,867 \| collagenIII-backbone-R \| cggaagacaaaagcttaTCCCGGGCTGCCAGCAG \|  \| \| 2,868 \| GPPG24 - backbone- R \| cggaagacaaaagcttaTCCGGGCGGACCCGG \|  \| \| 2,869 \| gppg6-backbone-R \| cggaagacaaaagcttaCCCTGGTGGTCCTCCTGGC \|  \| \| CRISPR oligonucleotides \| \| \|  \| \| 2,874 \| pyrG_sgRNA1-Cas12a \| atctacaacagtagaaattaGACTGCGGCATCGGATGTC \|  \| \| 2,875 \| pyrG_sgRNA2-Cas12a \| atctacaacagtagaaattaACCATAGTAGCCTGCAGGAT \|  \| \| 2,876 \| Crispr-arm1-fw \| ccgatgtggctggtgcgtggaggggctcgcgatgatttcccgggatgtcgagcacgggtaCGGATCCGGAGGCCCTTTCG \|  \| \| 2,877 \| Crispr12a-arm2-rev \| ggcacaaggatcaatgcggtacgacgatttgatgcagataagcaggctgcgaagtagtaaGCCGCCAGCTTGGCATCAGA \|  \| \| 2,878 \| Crispr12a-arm2-fw \| gcaaggaaactccgcctttgcaggtgtggctgaaccccacgggtcggaggcggagcaatcCGGATCCGGAGGCCCTTTCG \|  \| \| 2,879 \| Crispr-arm2-rev \| tgcgtagagaaaatggcgacgggtggctgataagggcggtgataagcttaattgtcatcgGCCGCCAGCTTGGCATCAGA \|  \| \| 2,946 \| pyrG sgRNA1 Cas12a \| gactgcggcatcggatgtcATCTACAACAGTAGAAATTA \|  \| \| 2,947 \| pyrG sgRNA2 Cas12a \| accatagtagcctgcaggatATCTACAACAGTAGAAATTA \|  \| \| 2,948 \| sgRNA1 pyrG cas12a FW \| AGATGACTGCGGCATCGGATGTC \|  \| \| 2,949 \| sgRNA1 pyrG cas12a Rev \| AAAAGACATCCGATGCCGCAGTC \|  \| \| 2,950 \| sgRNA2 pyrG cas12a FW \| AGATACCATAGTAGCCTGCAGGAT \|  \| \| 2,951 \| sgRNA2 pyrG cas12a Rev \| AAAAATCCTGCAGGCTACTATGGT \|  \| \| 2,966 \| sgRNA1 homology arm 3’ R \| ccgcattgatccttgtgccacaccatagtagcctgcaggatcaataccgtttggacatccGCCGCCAGCTTGGCATCAGA \|  \| \| 2,967 \| sgRNA2 homology arm 5' F \| cgagcacgggtagtcagactgcggcatcggatgtccaaacggtattgatcctgcaggctaCGGATCCGGAGGCCCTTTCG \|  \| \| 2,968 \| sgRNA2 homology arm 3' R \| agagttactacttcgcagcctgcttatctgcatcaaatcgtcgtaccgcattgatccttgGCCGCCAGCTTGGCATCAGA \|  \| \| 2,969 \| pyrG locus conf \| GGAAGTCCCGTATTTCTGCTGACG \|  \| \| 2,970 \| homology arm positive control pyrG \| ccgatgtggctggtgcgtggaggggctcgcgatgatttcccgggatgtcgagcacgggtaGGGAGCTGTTGGCTGGCTGG \|  \| \| 3,062 \| human P4H rev \| cggaagacaaaagCTTAAAGTTCGTCTTTTACAGCCTTCTGATCG \|  \| \| 3,063 \| plant P4H new R \| cggaagacaaaagcttaTGACGTCGCCTTCTGGCG \|  \| \| 3,064 \| viral P4H new R \| cggaagacaaaagcttaCTTCACTGCTCTGATCCATTGGTTGGC \|  \| \| 3,125 \| sgRNA1prtT \| tccgttgagtatcccatccgATCTACAACAGTAGAAATTA \|  \| \| 3,126 \| sgRNA2prtT \| actcgaaccgtggacgagatATCTACAACAGTAGAAATTA \|  \| \| 3,127 \| prtT fw \| AATCCCATTCCCAGGGTTGG \|  \| \| 3,128 \| prtT R \| CTGATGGATCCGGTGGTGG \|  \| \| 3,129 \| prtTLeu112confirm \| TCCAAGACAGTGCTGCGATG \|  \| \| 3,230 \| prtTsequence-R \| CAGTCGAATGCGGTTGGCG \|  \| \| 3,318 \| colIIIfusionR \| CGGAAGACAAACTCCCGGGCTGCCAGC \|  \| \| 3,319 \| colIIIfusionF \| CGGAAGACAAGAGTGAGCAAGGGCGAGGAGC \|  \| \| 3,328 \| ORFprtT-fw \| ATGACTCGAACCGTGGACGA \|  \| \| 3,334 \| ORFprtT-R \| TTACAGCGTCGCTGACCGAT \|  \| \| 3,364 \| flankprtT-fw \| ACGTGATGTTTCGTGGCACG \|  \| \| 3,365 \| flankprtT-r \| AGCGTCTACCCCAGGTCAAG \|  \| \| 3,366 \| lgflankprtT-fw \| GGGCAAGCGAGATGAACAACAC \|  \| \| 3,367 \| lgflankprtT-r \| CCTTGTACTTTGGAGCCGGCG \|  \| \| 3,452 \| sgRNAprtT3 \| ggctcgcatctgaggttctgATCTACAACAGTAGAAATTA \|  \| \| 3,453 \| sgRNAprtT4 \| aggagcgcctcacctccctaATCTACAACAGTAGAAATTA \|  \| \| 3,949 \| qPCR1-colIII-F \| CTGGGCTGAAAGGAGAGAAC \|  \| \| 3,950 \| qPCR1-colIII-R \| GTGCTCCATCATTCCCTCTG \|  \| \| 3,951 \| qPCR2-colIII-F \| GCCTGGATTTCCCGGAATGAA \|  \| \| 3,952 \| qPCR2-colIII-R \| GAGCCCGTTCTCTCCTTTCAG \|  \| \| 4,007 \| HiBiT R \| CGGAAGACAAAAGCGCTAATCTTCTTGAACAGGCGCCAGCCGGACACTCCCGGGCTGCCAGC \|  \| \| 4,020 \| qPCR1 An09g03780pepD FW \| TTTGACGAGGGTGTGCTCTC \|  \| \| 4,021 \| qPCR1 An09g03780pepD R \| GTTTGAGAATGACGCACGGG \|  \| \| 4,050 \| humanP4H qPCR1 F \| GAGTGGTCGGAGCTGGAAAA \|  \| \| 4,051 \| humanP4H qPCR1 R \| GGTGTAAGCGACCTTCCCAA \|  \| \| 4,052 \| humanP4H qPCR2 F \| TGCACCCTGGATTCTTCACC \|  \| \| 4,053 \| humanP4H qPCR2 R \| CGTTGACAGGATGACCCACA \|  \| \| 4,081 \| alpha amylase secretion signal colIII \| cggaagacaaaatggtcgcgtggtggtccctatttctgtacggccttcaggtcgcggcacctgctttggctGGTTATCCTGGTCCCGCCGGACCG \|  \| \| 4,082 \| pgxA secretion signal col III \| cggaagacaaaatgagactcacgcacgttctctctcacacgcttggccttcttgcgctaggagcaacagcagaagctGGTTATCCTGGTCCCGCCG \|  \| \| 4,083 \| pgaI secretion signal col III \| cggaagacaaaatgcactcttaccagcttcttggcctggccgctgtcggctccctcgtctctgccGGTTATCCTGGTCCCGCCGGACCGCC \|  \| \| 4,113 \| hacA-qPCR-fw \| CAGCTTCTCCTACCCTAACTCCT \|  \| \| 4,114 \| hacA-qPCR-R \| ACGTCAAAGAGAGAGAGGGCAC \|  \| \| 4,115 \| pyrG donor DNA fw \| ccgatgtggctggtgcgtggaggggctcgcgatgatttcccgggatgtcgagcacgggtaGTGCCGAATTCGGATCCGGAG \|  \| \| 4,121 \| An15g03420-conf-fw-2 \| CCTCTCCAAAGTCTATTCAGTCCCTCG \|  \| \| 4,122 \| An15g03420-conf-fw3 \| GCCATATGGCCTGATCTGGTGGC \|  \| \| 4,276 \| PhttA-TU1-fw \| gtggtctcaggagATTGAAAGATTTTGAGATTAGGTGTGAAAGGTG \|  \| \| 4,277 \| PhttA-TU1-r \| gtggtctcaagcgTTTGTTGGATGGTATGTGAGGTTTTTT \|  \| \| 4,278 \| gaaA-HDR-fw \| tgtgattgctgtggtgtaaatttaactaagaaaagatattaagaGGAGATTGAAAGATTTTGAGATTAGGTGTGAAAGGTG \|  \| \| 4,279 \| gaaA-HDR-r \| gcaaaacccggtccagcaggatttcttatatgaccgccaacccactgagcgaccagattATGTGCATCCTCTAGTAGCGAGG \|  \| \| 4,280 \| PgaaA-sgRNAcas12a \| agaatatgacaagaaagaagATCTACAACAGTAGAAATTA \|  \| \| 4,297 \| HDR-FW An08g04490 \| tgtgcccaagcctatctctcggccagcttcgagtaagtcggctgcgactacgggtgaggcGAAGACAAGCAAGAATTCAAGCTTGGAG \|  \| \| 4,298 \| HDR-R An08g04490 \| tatacaagtccgagcaatggaacccgcccggaataatctgcacgggttcgttcgccgtgcCCACTTCGTGCAGAAGACAATAGTAGCG \|  \| \| 4,299 \| sgRNA-16-An08g04490 \| gtgatggtccagcagctgctATCTACAACAGTAGAAATTA \|  \| \| 4,300 \| sgRNA-17 An08g04490 \| accagcggaccaccgggccgATCTACAACAGTAGAAATTA \|  \| \| 4,301 \| An08g04490-conf-fw \| ATGCGTTCCTTCTCCGTTGTCG \|  \| \| 4,302 \| An08g04490-conf-r \| TCAAGCATAATACTCCTCCACCCAC \|  \| \| 4,303 \| HDR-FW-An04g07220 \| tcctcgacttcgatccatcccaactcaccgtgactacgacctcagccgatgcgcttcttgGAAGACAAGCAAGAATTCAAGCTTGGAG \|  \| \| 4,304 \| HDR-R An04g07220 \| cagacctcagaacagcctgtgaactacccccgatcctagccgccgacgacgtagtcattgCCACTTCGTGCAGAAGACAATAGTAGCG \|  \| \| 4,305 \| sgRNA-18 An04g07220 \| tggtagccttttgaggccgcATCTACAACAGTAGAAATTA \|  \| \| 4,306 \| gRNA-19 An04g07220 \| gatacacctctcctattcccATCTACAACAGTAGAAATTA \|  \| \| 4,307 \| An04g07220-conf-FW \| ATGGAACCAATCATCCATCCCATATTCG \|  \| \| 4,308 \| An04g07220-conf-R \| CCTCCGCATAACCATCCTTAACCT \|  \| \| 4,309 \| An12g08560-HDR-Fw \| atgcctccggatgcaaaatcgcctggctaccagcctggtatggcagtattaccGAAGACAAGCAAGAATTCAAGCTTGGAG \|  \| \| 4,310 \| An12g08560-HDR-R \| gaactcgctcagcgaatgcaaggcggacatcaattctgtgtcacctggcgacatgacctgCCACTTCGTGCAGAAGACAATAGTAGCG \|  \| \| 4,311 \| sgRNA-20 An12g08560 \| taggccacatcctgccaaggATCTACAACAGTAGAAATTA \|  \| \| 4,312 \| sgRNA-21 An12g08560 \| tctctcatatccggcaagacATCTACAACAGTAGAAATTA \|  \| \| 4,313 \| An1208560-conf-F \| ATGCCTCCGGATGCAAAATCG \|  \| \| 4,314 \| An1208560-conf-R \| TCACGCGCCTAGCAGATGG \|  \| \| 4,315 \| An04g07720-HDR-Fw \| tcctcgacttcgatccatcccaactcaccgtgactacgacctcagccgatgcgcttcttgCCCCACTCTGTGAAGACAAGC \|  \| \| 4,316 \| An08g04480-HDR-fw \| tgtgcccaagcctatctctcggccagcttcgagtaagtcggctgcgactacgggtgaggcGGCCCCACTCTGTGAAGACAAGC \|  \| \| 4,317 \| An12g08560-HDR-fw-2 \| atgcctccggatgcaaaatcgcctggctaccagcctggtatggcagtattaccGGCCCCACTCTGTGAAGACAAGC \|  \| \| 4,342 \| An02g07710-confirm-fw \| GGCTGTACCACCGACGTAGC \|  \| \| 4,343 \| An02g07710-confirm-R \| GAACGGCGTGCTTGCGAATG \|  \| \| 4,362 \| An02g07710-conf-fw2 \| GTCGGAGGTGTTGTCGGCG \|  \| \| 4,363 \| An02g07710-conf-R2 \| CGTTGGAGCCCATGCAGACG \|  \| \| 4,614 \| 4614-rpl-fw \| GTGGTCTCAGGAGATTTCACATTTCCTG \|  \| \| 4,615 \| 4615-rpl-rev \| GTGGTCTCAAGCGCTGGACGGAAATCGGAATATCAGTCTCTG \|  \| \| 4,616 \| An14g04280-HDR-fw \| tgtgataaatattcgaatgacctccgggcagtgagactctcggcttctcctttgattctcTGCCGATGTATCACCTCTCGC \|  \| \| 4,617 \| HDR-An14g04280-R \| tgtggaagaattgtccagagactggtaggcaattgacaattgatcctagcagcacaGCTGGACGGAAATCGGAATATCAGTCTCTG \|  \| \| 4,618 \| sgRNA-30-template \| acagggcattcccaggggaaATCTACAACAGTAGAAATTA \|  \| \| 4,619 \| An14g04280-conf-fw \| CAGTCGCAGTCGAGTCGGTCTC \|  \| \| 4,620 \| An14g04280-conf-r \| CGAGTAGGCCACTGCTGTGAGG \|  \| \| 4,694 \| qPCR-gaaA-f \| AGGACACGATTACTCTACTTG \|  \| \| 4,695 \| qPCR-gaaA-r \| GAGCCCATATAATGGAAGTAC \|  \| \| 4,696 \| qPCR-gatA-fw-1 \| CGAGGCCGAGTTGCTGGAAT \|  \| \| 4,697 \| qPCR-gatA-r-1 \| CTGGACAGCGACTGGGACAC \|  \| \| 4,698 \| qPCR-gatA-fw-2 \| ATGGTGGCCGTGTCATAGCG \|  \| \| 4,699 \| qPCR-gatA-r-2 \| ACTCGGCCTCGAATGCTTGG \|  \| \| 4,706 \| HDR-fw-pyrG \| ttcgagaccatttgctcaacatcacccttgcccgactcgctacttattaccccggggtccACAATTACGAATTCCCATGGGGAG \|  \| \| 4,707 \| HDR-r-pyrG \| gagcaaggaggatcagaagaggggacaaaaataacaaattcacaggatatcccgtccatcCGTGCAGAAGACAATCTGAGCG \|  \| \|  \|  \| **Other primers used not designed from this study** \|  \| \| 2495 \| Luc_R_1 \| CGGAAGACAATTCATCTTCGTCCCAGTAAGCTATGTCTCC \|  \| \| 1336 \| pyrG*/XbaI-fw \| atctccactcgacctgcaggTCGCGGTCGTTTGTACGGCA \|  \| \| 2242 \| pTET-7x-R \| CGGAAGACAACATTGGTGATGTCTGCTCAAGCGGG \|  \| \| 2729 \| AorPyrG-Rev-2 \| CGGAAGACAAAAGCTTATTGCGCACCAACACGCTTCAG \|  \| \| 2452 \| thxK_FW \| CGGAAGACAAGCTTTGACCTGGCGCACATAAATATGAATTCTTTGG \|  \| \| 2739 \| pAoPyrG-Rev \| CGGAAGACAACATTGTTGGCGATGGAGGGGTAGC \|  \| \| 2728 \| AorPyrG-Fw-2 \| CGGAAGACAACGTTTTCACGACAGGTGTCAACCTC \|  \| |
| --- | --- | --- | --- | --- | --- | --- | --- | --- | --- | --- | --- | --- | --- | --- | --- | --- | --- | --- | --- | --- | --- | --- | --- | --- | --- | --- | --- | --- | --- | --- | --- | --- | --- | --- | --- | --- | --- | --- | --- | --- | --- | --- | --- | --- | --- | --- | --- | --- | --- | --- | --- | --- | --- | --- | --- | --- | --- | --- | --- | --- | --- | --- | --- | --- | --- | --- | --- | --- | --- | --- | --- | --- | --- | --- | --- | --- | --- | --- | --- | --- | --- | --- | --- | --- | --- | --- | --- | --- | --- | --- | --- | --- | --- | --- | --- | --- | --- | --- | --- | --- | --- | --- | --- | --- | --- | --- | --- | --- | --- | --- | --- | --- | --- | --- | --- | --- | --- | --- | --- | --- | --- | --- | --- | --- | --- | --- | --- | --- | --- | --- | --- | --- | --- | --- | --- | --- | --- | --- | --- | --- | --- | --- | --- | --- | --- | --- | --- | --- | --- | --- | --- | --- | --- | --- | --- | --- | --- | --- | --- | --- | --- | --- | --- | --- | --- | --- | --- | --- | --- | --- | --- | --- | --- | --- | --- | --- | --- | --- | --- | --- | --- | --- | --- | --- | --- | --- | --- | --- | --- | --- | --- | --- | --- | --- | --- | --- | --- | --- | --- | --- | --- | --- | --- | --- | --- | --- | --- | --- | --- | --- | --- | --- | --- | --- | --- | --- | --- | --- | --- | --- | --- | --- | --- | --- | --- | --- | --- | --- | --- | --- | --- | --- | --- | --- | --- | --- | --- | --- | --- | --- | --- | --- | --- | --- | --- | --- | --- | --- | --- | --- | --- | --- | --- | --- | --- | --- | --- | --- | --- | --- | --- | --- | --- | --- | --- | --- | --- | --- | --- | --- | --- | --- | --- | --- | --- | --- | --- | --- | --- | --- | --- | --- | --- | --- | --- | --- | --- | --- | --- | --- | --- | --- | --- | --- | --- | --- | --- | --- | --- | --- | --- | --- | --- | --- | --- | --- | --- | --- | --- | --- | --- | --- | --- | --- | --- | --- | --- | --- | --- | --- | --- | --- | --- | --- | --- | --- | --- | --- | --- | --- | --- | --- | --- | --- | --- | --- | --- | --- | --- | --- | --- | --- | --- | --- | --- | --- | --- | --- | --- | --- | --- | --- | --- | --- | --- | --- | --- | --- | --- | --- | --- | --- | --- | --- | --- | --- | --- | --- | --- | --- | --- | --- | --- | --- | --- | --- | --- | --- | --- | --- | --- | --- | --- | --- | --- | --- | --- | --- | --- | --- | --- | --- | --- | --- | --- | --- | --- | --- | --- | --- | --- | --- | --- | --- | --- | --- | --- | --- | --- | --- | --- | --- | --- | --- | --- | --- | --- | --- | --- | --- | --- | --- | --- | --- | --- | --- | --- | --- | --- | --- | --- | --- | --- | --- | --- | --- | --- | --- | --- | --- | --- | --- | --- | --- | --- | --- | --- | --- | --- | --- | --- | --- | --- | --- | --- | --- | --- | --- | --- | --- | --- | --- | --- | --- | --- | --- | --- | --- | --- | --- | --- | --- | --- | --- | --- | --- | --- | --- | --- | --- | --- | --- | --- | --- | --- | --- | --- | --- | --- | --- | --- | --- | --- | --- | --- | --- | --- | --- | --- | --- | --- | --- | --- | --- | --- | --- | --- | --- | --- | --- | --- | --- | --- | --- | --- | --- | --- | --- | --- | --- | --- | --- | --- | --- | --- | --- | --- | --- | --- | --- | --- | --- | --- | --- | --- | --- | --- | --- | --- | --- | --- | --- | --- | --- | --- | --- | --- | --- | --- | --- | --- | --- | --- | --- | --- | --- | --- | --- | --- | --- | --- | --- | --- | --- | --- | --- | --- | --- | --- | --- | --- | --- | --- | --- | --- | --- | --- | --- | --- | --- | --- | --- | --- | --- | --- | --- | --- | --- | --- | --- | --- | --- | --- | --- | --- | --- | --- | --- | --- | --- | --- | --- | --- | --- | --- | --- | --- | --- | --- | --- | --- | --- | --- | --- | --- | --- | --- | --- | --- | --- | --- | --- | --- | --- | --- | --- | --- | --- | --- | --- | --- | --- |
