# Supplementary material for: The multipurpose cell factory Aspergillus niger can be engineered to produce hydroxylated collagen
Source: Biotechnol Biofuels Bioprod. 2025 Aug 8;18:88. doi: 10.1186/s13068-025-02681-y (PMC12333218; doi:10.1186/s13068-025-02681-y)
Supplement: Supplementary file 3 — Additional file 3. Recombinant Protein sequences. [file 13068_2025_2681_MOESM3_ESM.pptx]

## Slide 1
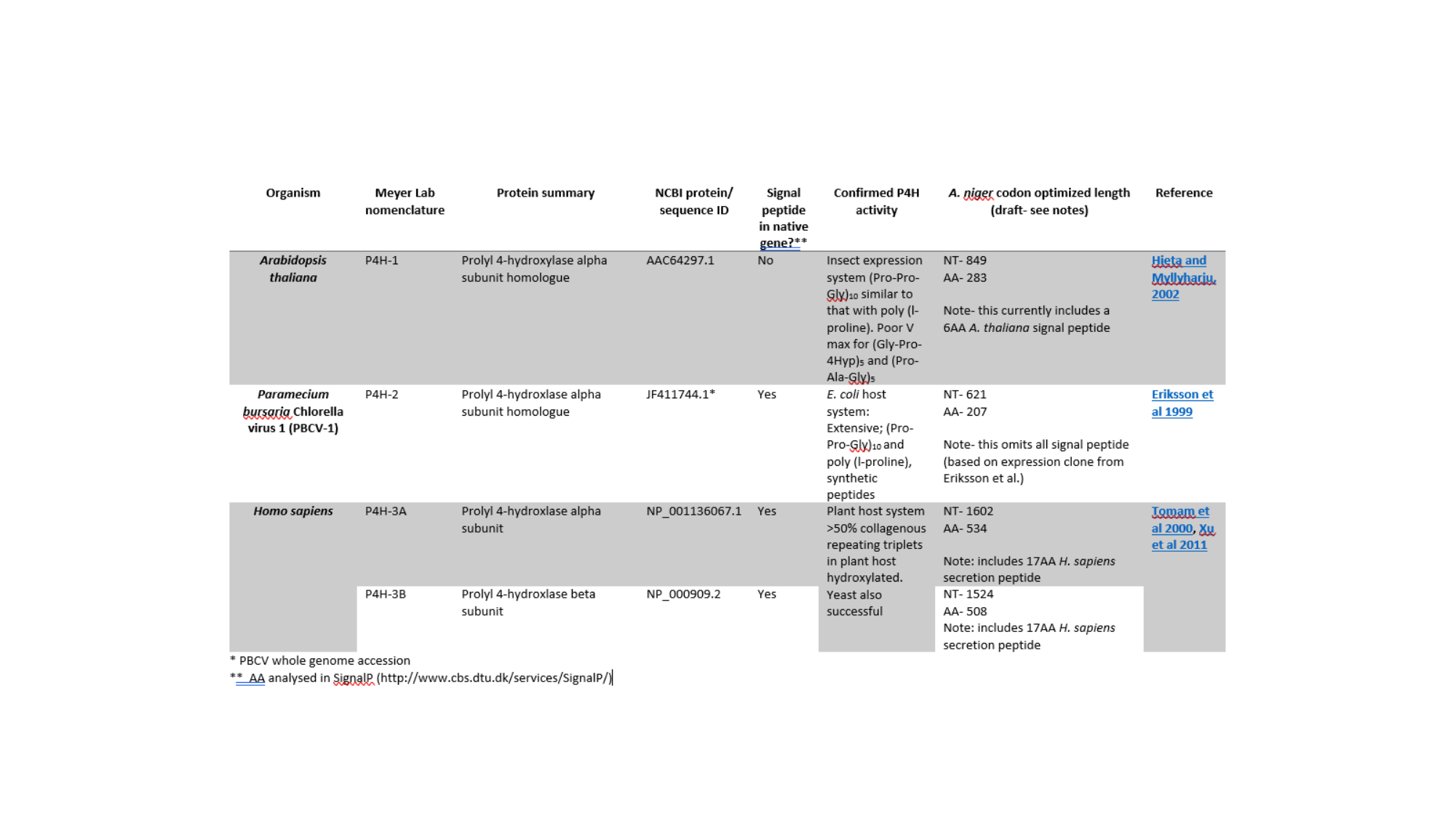

## Slide 2
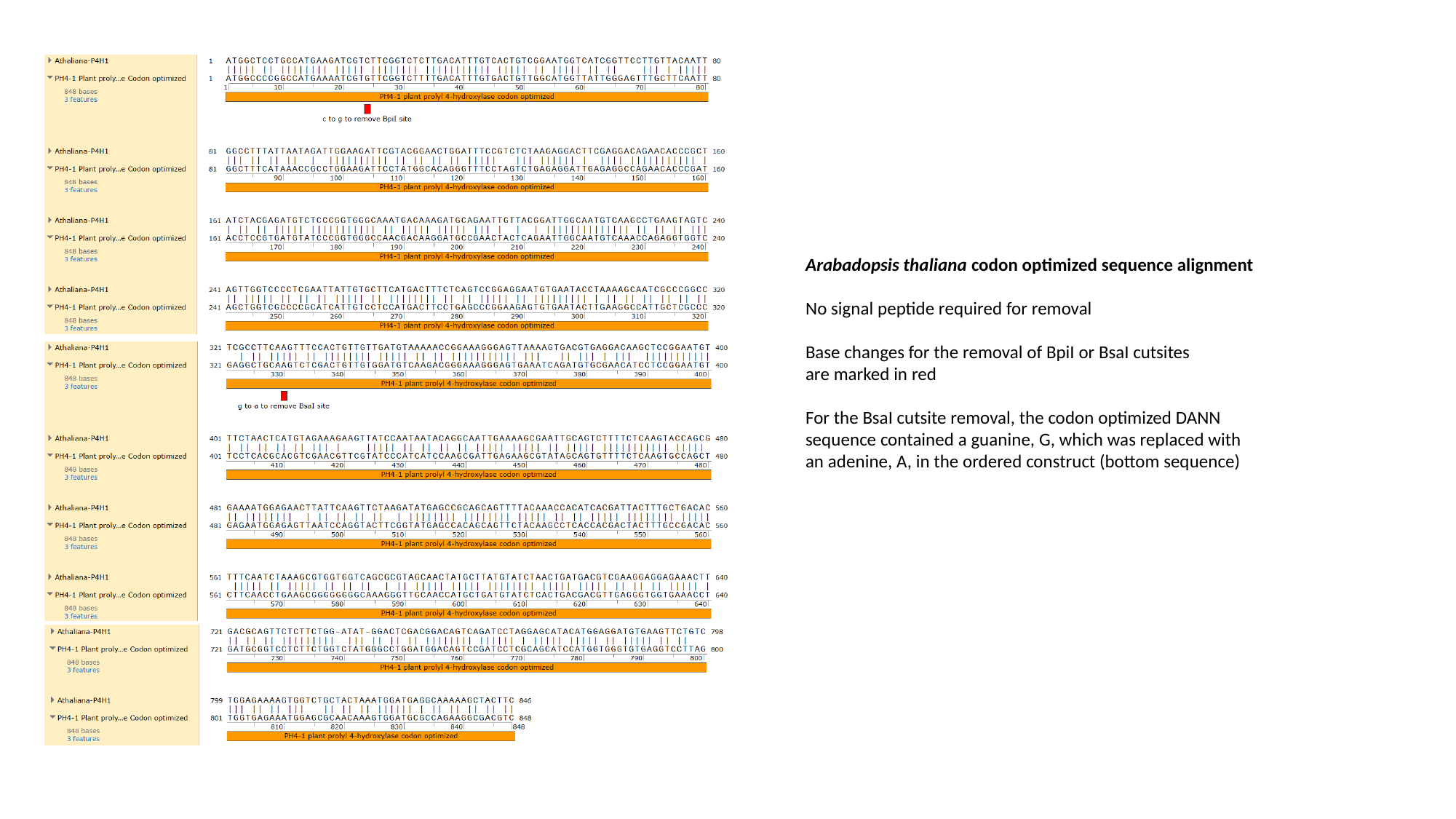

Arabadopsis thaliana codon optimized sequence alignment
No signal peptide required for removal
Base changes for the removal of BpiI or BsaI cutsites
are marked in red
For the BsaI cutsite removal, the codon optimized DANN
sequence contained a guanine, G, which was replaced with
an adenine, A, in the ordered construct (bottom sequence)

## Slide 3
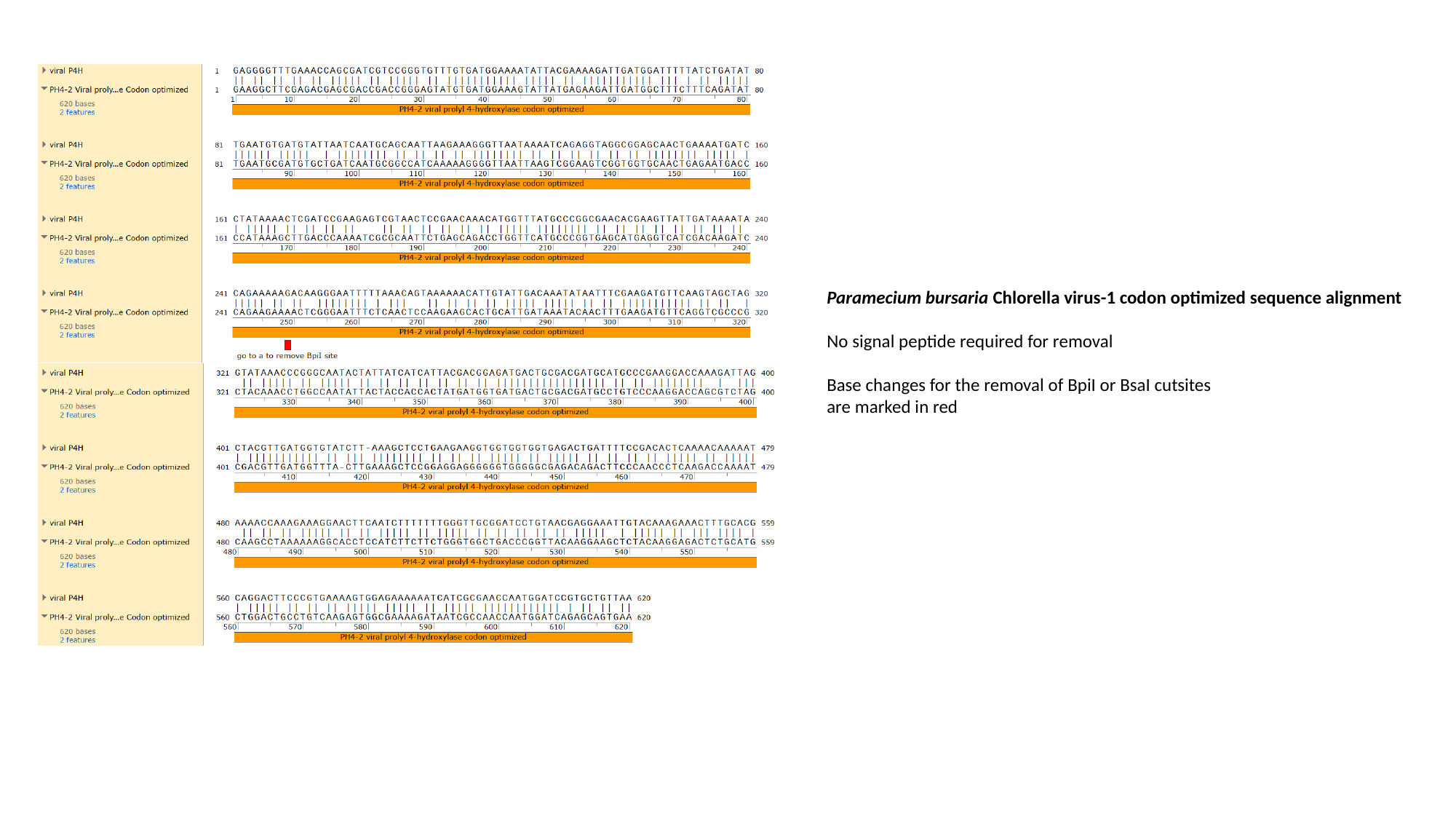

Paramecium bursaria Chlorella virus-1 codon optimized sequence alignment
No signal peptide required for removal
Base changes for the removal of BpiI or BsaI cutsites
are marked in red

## Slide 4
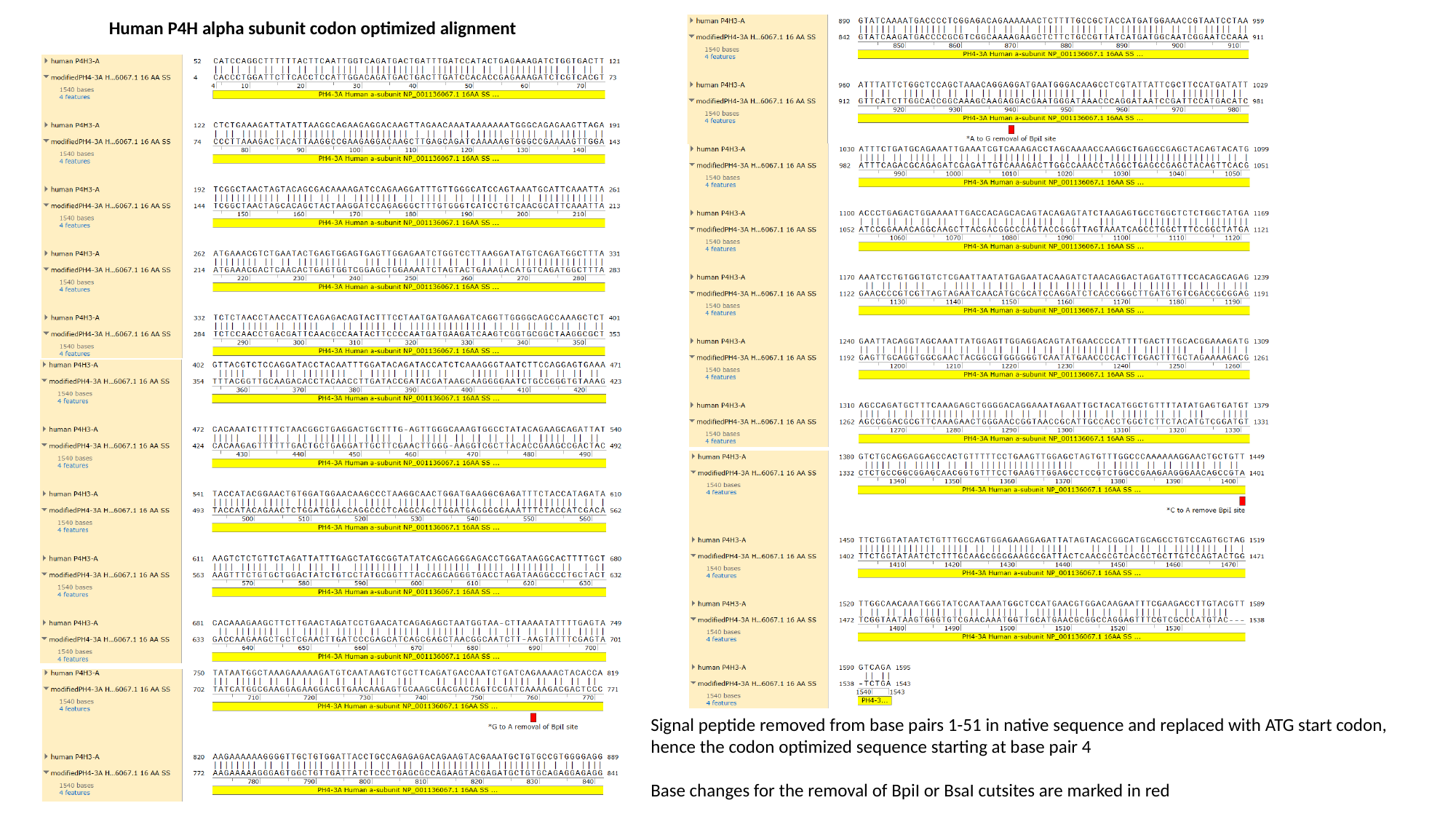

Human P4H alpha subunit codon optimized alignment
Signal peptide removed from base pairs 1-51 in native sequence and replaced with ATG start codon, hence the codon optimized sequence starting at base pair 4
Base changes for the removal of BpiI or BsaI cutsites are marked in red

## Slide 5
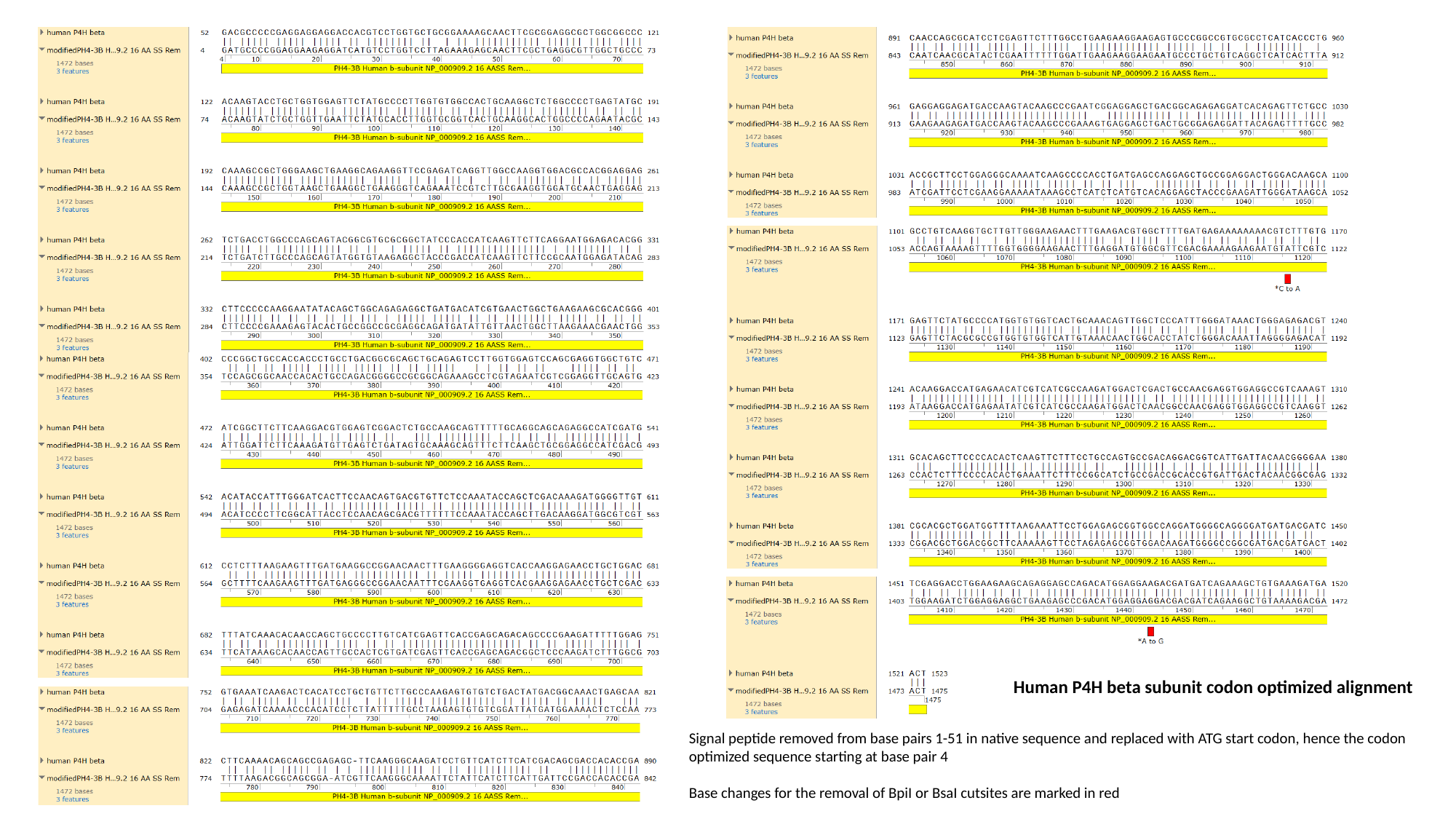

Human P4H beta subunit codon optimized alignment
Signal peptide removed from base pairs 1-51 in native sequence and replaced with ATG start codon, hence the codon optimized sequence starting at base pair 4
Base changes for the removal of BpiI or BsaI cutsites are marked in red

## Slide 6
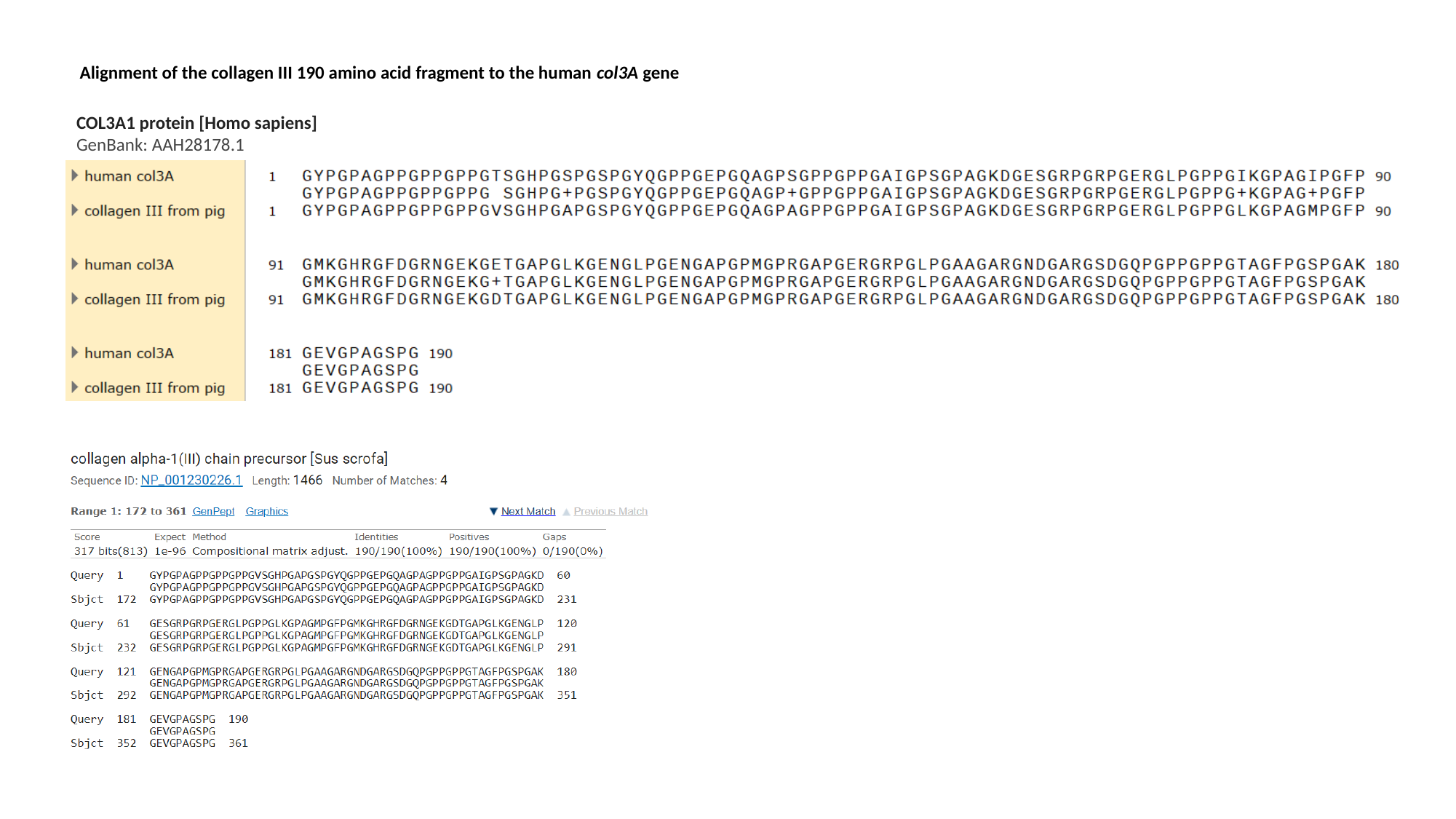

Alignment of the collagen III 190 amino acid fragment to the human col3A gene
COL3A1 protein [Homo sapiens]
GenBank: AAH28178.1
