# Supplementary material for: The multipurpose cell factory Aspergillus niger can be engineered to produce hydroxylated collagen
Source: Biotechnol Biofuels Bioprod. 2025 Aug 8;18:88. doi: 10.1186/s13068-025-02681-y (PMC12333218; doi:10.1186/s13068-025-02681-y)
Supplement: Supplementary file 4 — Additional file 4. PCR proofs of cassette integration at the target locus. [file 13068_2025_2681_MOESM4_ESM.pptx]

## Slide 1
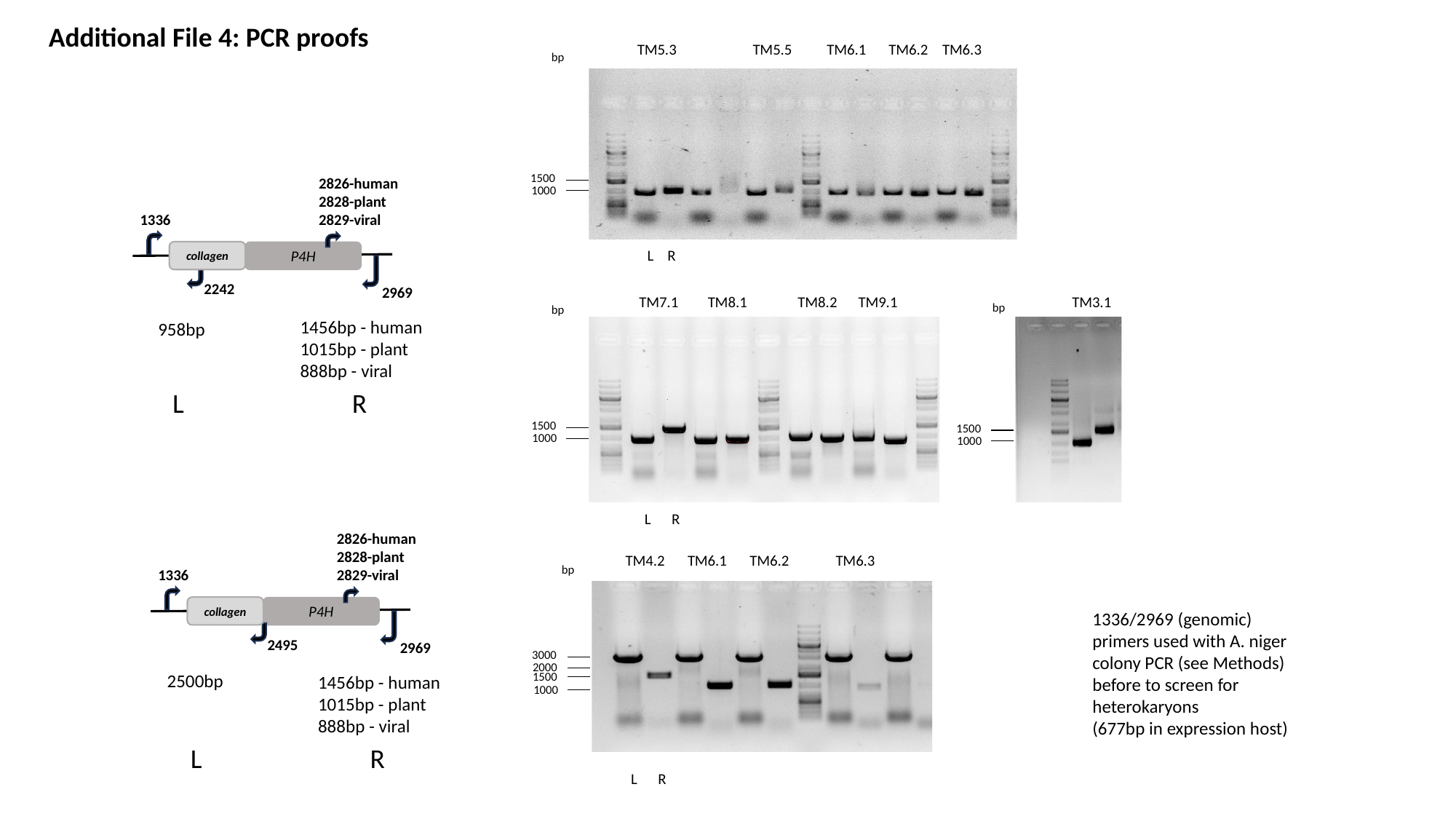

Additional File 4: PCR proofs
TM5.3
TM5.5
TM6.1
TM6.2
TM6.3
bp
1500
2826-human
2828-plant
2829-viral
1000
1336
L R
collagen
P4H
2242
2969
TM7.1
TM8.1
TM8.2
TM9.1
TM3.1
bp
bp
1456bp - human
1015bp - plant
888bp - viral
958bp
L
R
1500
1500
1000
1000
L R
2826-human
2828-plant
2829-viral
TM4.2
TM6.1
TM6.2
TM6.3
bp
1336
collagen
P4H
1336/2969 (genomic)
primers used with A. niger colony PCR (see Methods)
before to screen for heterokaryons
(677bp in expression host)
2495
2969
3000
2000
1500
2500bp
1456bp - human
1015bp - plant
888bp - viral
1000
L
R
L R

## Slide 2
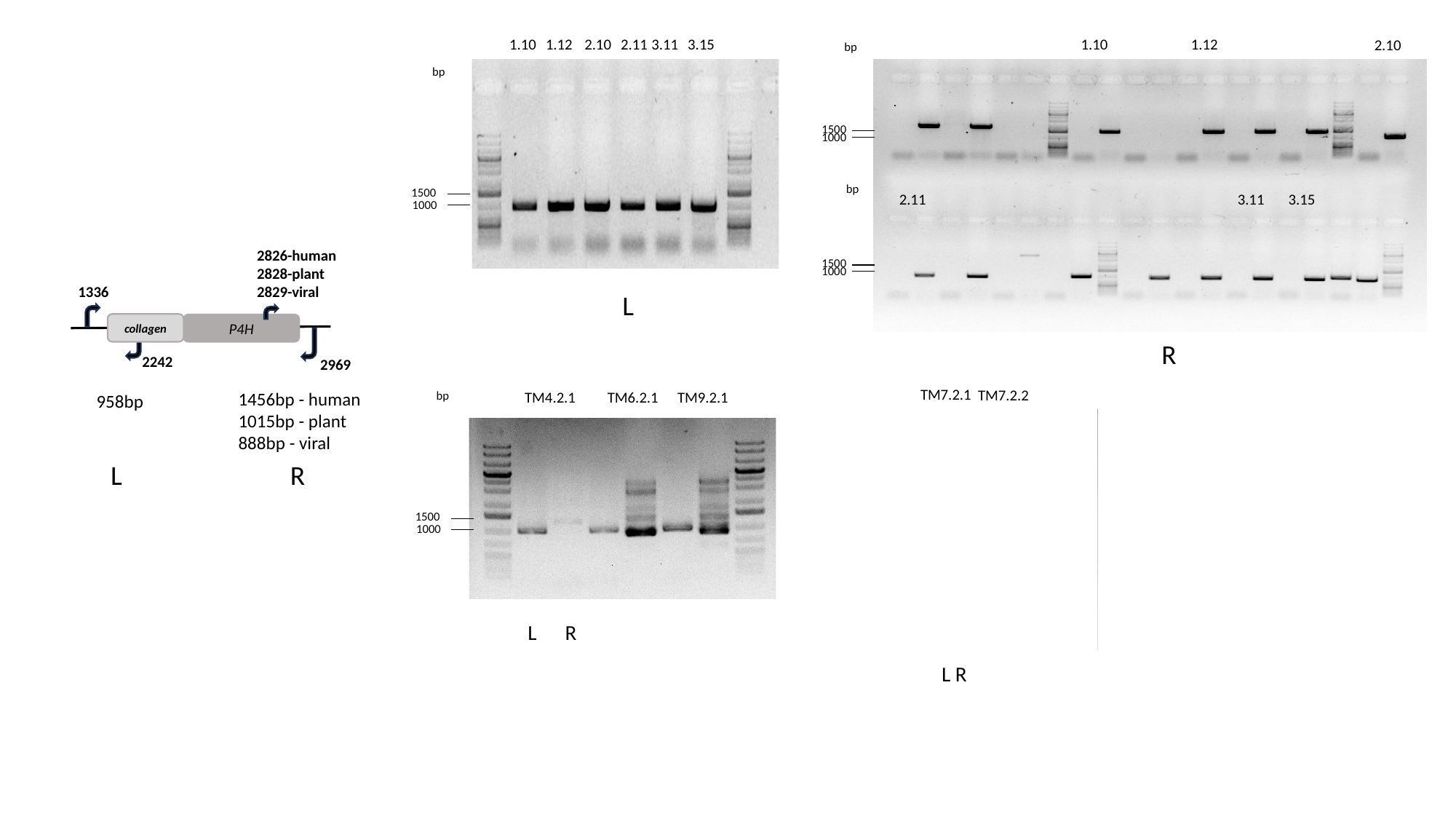

1.10
1.12
2.10
2.11
3.11
3.15
1.10
1.12
2.10
bp
bp
1500
1000
bp
1500
2.11
3.15
3.11
1000
2826-human
2828-plant
2829-viral
1500
1000
1336
L
collagen
P4H
R
2242
2969
TM7.2.1
TM7.2.2
1456bp - human
1015bp - plant
888bp - viral
TM4.2.1
TM6.2.1
TM9.2.1
bp
958bp
L
R
1500
1000
L R
L R

## Slide 3
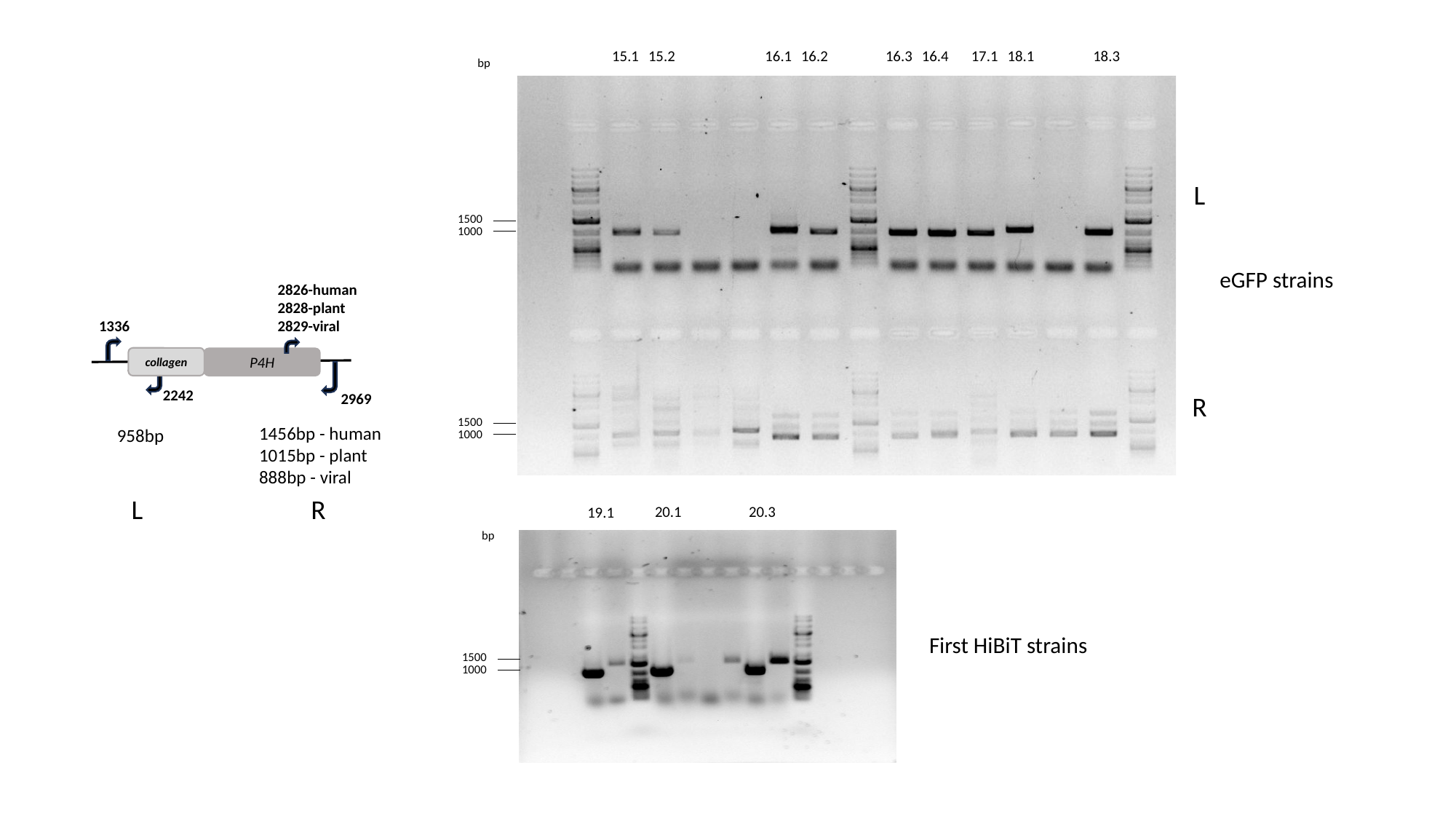

15.1
15.2
16.1
16.2
16.3
16.4
17.1
18.1
18.3
bp
L
1500
1000
eGFP strains
2826-human
2828-plant
2829-viral
1336
collagen
P4H
2242
2969
R
1500
1456bp - human
1015bp - plant
888bp - viral
958bp
1000
L
R
20.3
20.1
19.1
bp
First HiBiT strains
1500
1000

## Slide 4
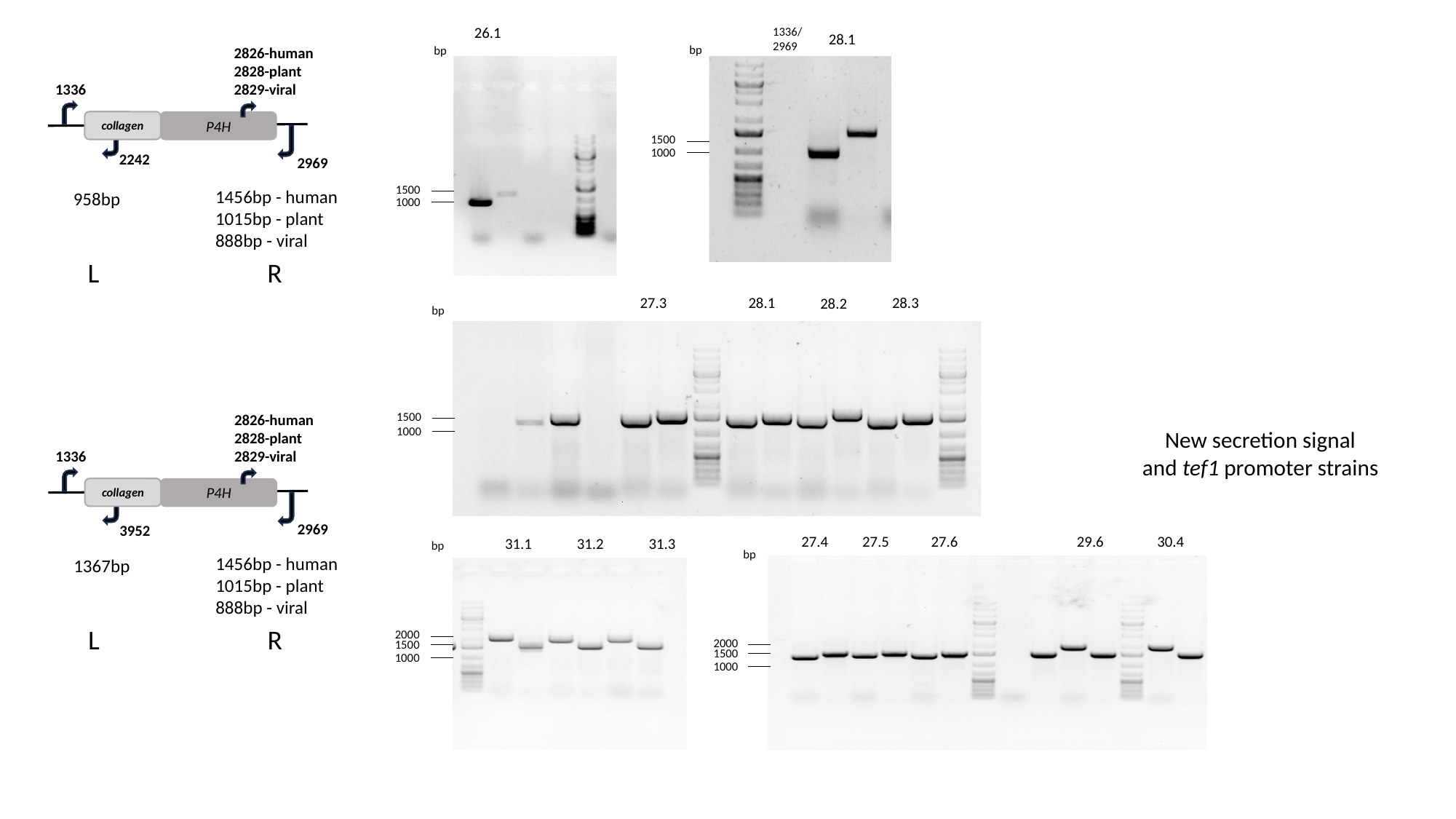

26.1
1336/
2969
28.1
bp
bp
2826-human
2828-plant
2829-viral
1336
collagen
P4H
1500
1000
2242
2969
1500
1456bp - human
1015bp - plant
888bp - viral
958bp
1000
L
R
27.3
28.1
28.3
28.2
bp
1500
2826-human
2828-plant
2829-viral
1000
New secretion signal
and tef1 promoter strains
1336
collagen
P4H
2969
3952
27.4
27.5
27.6
29.6
30.4
31.1
31.2
31.3
bp
bp
1456bp - human
1015bp - plant
888bp - viral
1367bp
L
R
2000
2000
1500
1500
1000
1000

## Slide 5
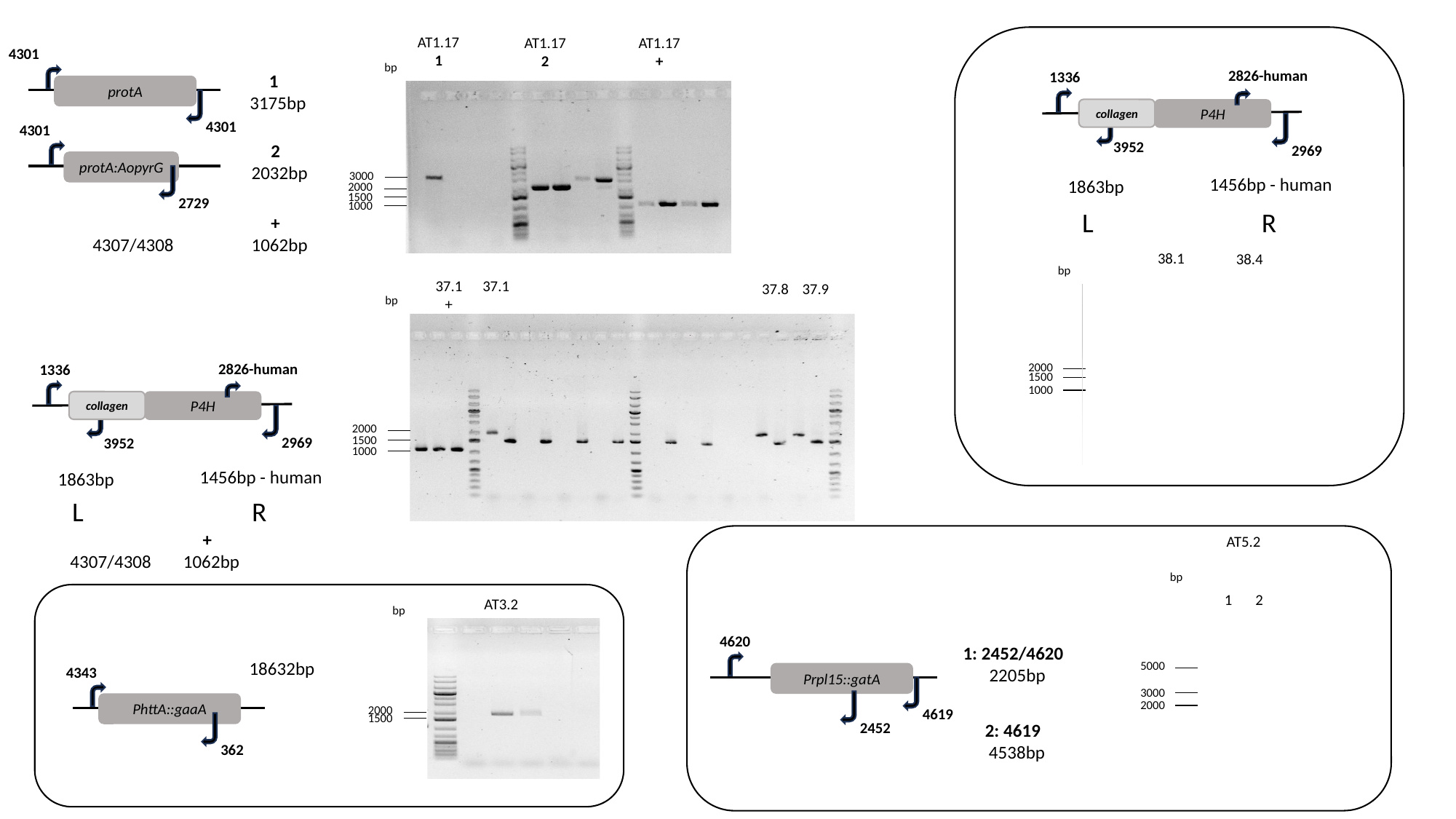

AT1.17
1
AT1.17
2
AT1.17
+
4301
bp
2826-human
1336
1
3175bp
protA
collagen
P4H
4301
4301
3952
2
2032bp
2969
protA:AopyrG
3000
1456bp - human
1863bp
2000
1500
2729
1000
L
R
+
1062bp
4307/4308
38.1
38.4
bp
37.1
37.1
+
37.8
37.9
bp
2000
2826-human
1336
1500
1000
collagen
P4H
2000
1500
2969
3952
1000
1456bp - human
1863bp
L
R
+
1062bp
AT5.2
4307/4308
bp
2
1
AT3.2
bp
4620
18632bp
1: 2452/4620
2205bp
5000
4343
Prpl15::gatA
3000
2000
PhttA::gaaA
2000
4619
1500
2452
2: 4619
4538bp
362

## Slide 6
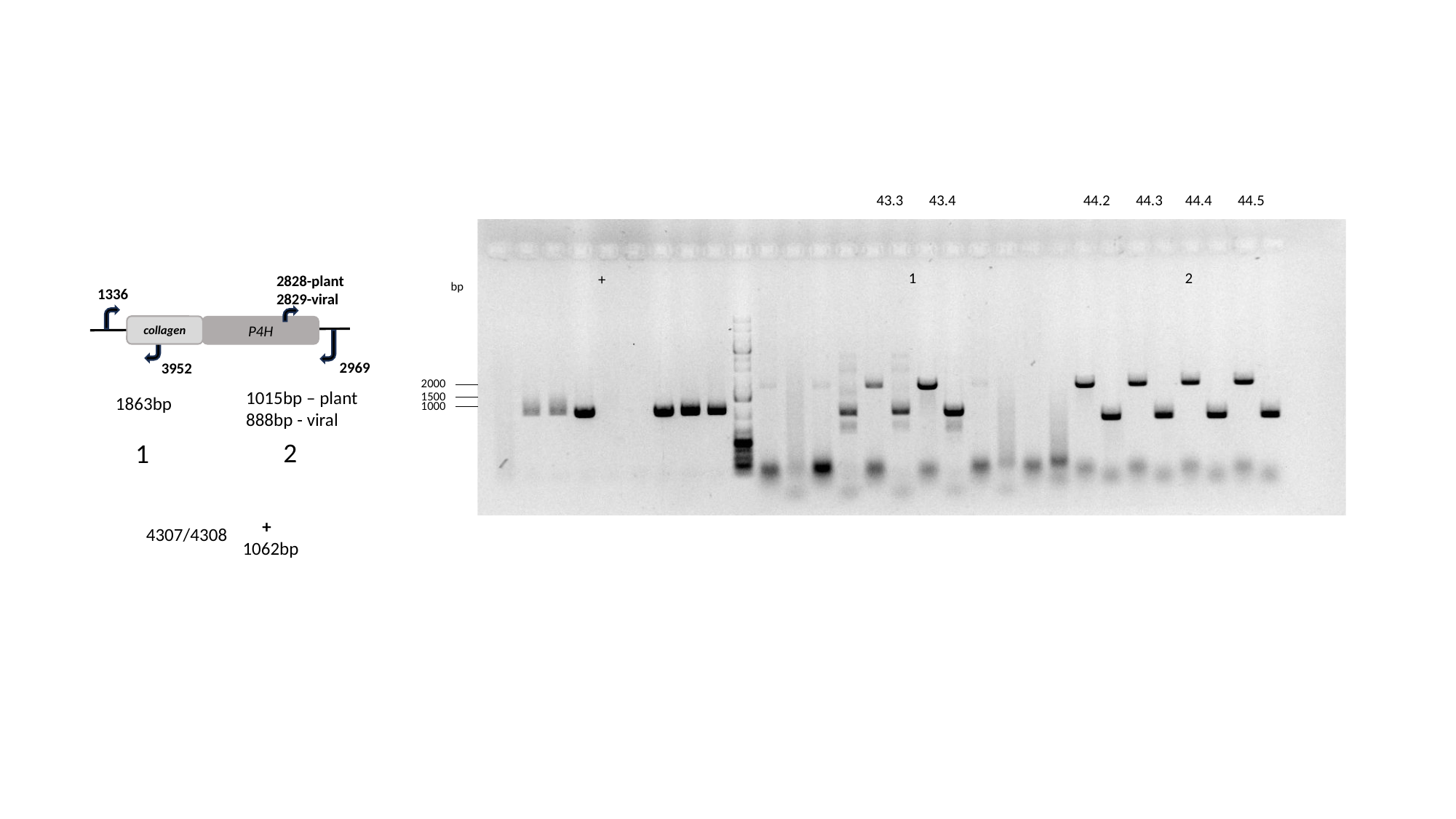

44.4
44.5
43.3
43.4
44.2
44.3
2
1
+
2828-plant
2829-viral
bp
1336
collagen
P4H
2969
3952
2000
1015bp – plant
888bp - viral
1500
1863bp
1000
2
1
+
1062bp
4307/4308

## Slide 7
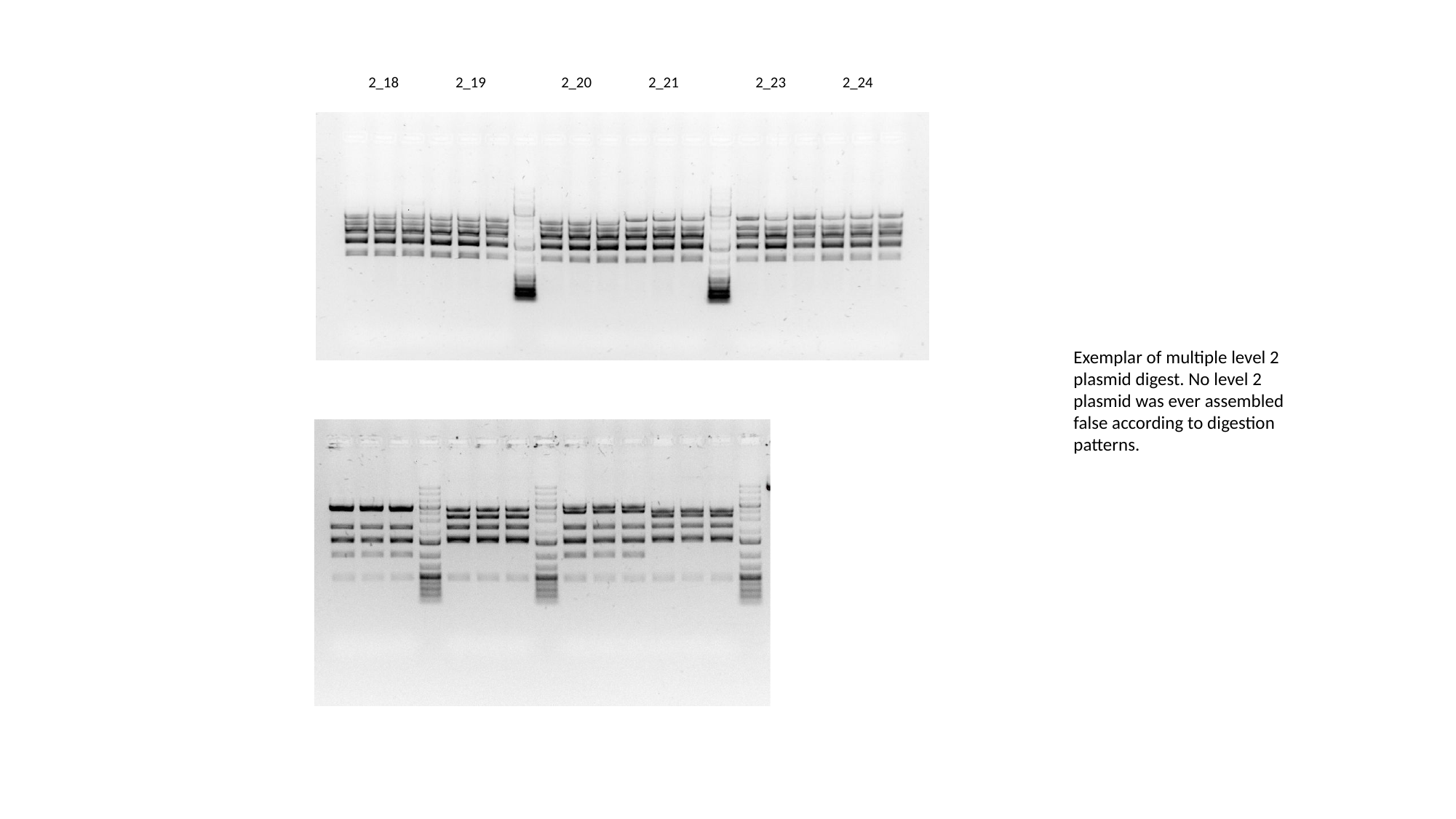

2_18
2_19
2_20
2_21
2_23
2_24
Exemplar of multiple level 2 plasmid digest. No level 2 plasmid was ever assembled false according to digestion patterns.
