# Supplementary material for: The multipurpose cell factory Aspergillus niger can be engineered to produce hydroxylated collagen
Source: Biotechnol Biofuels Bioprod. 2025 Aug 8;18:88. doi: 10.1186/s13068-025-02681-y (PMC12333218; doi:10.1186/s13068-025-02681-y)
Supplement: Supplementary file 5 — Additional file 5. SDS-PAGE of total proteins from A. niger isolates expressing collagen. [file 13068_2025_2681_MOESM5_ESM.pptx]

## Slide 1
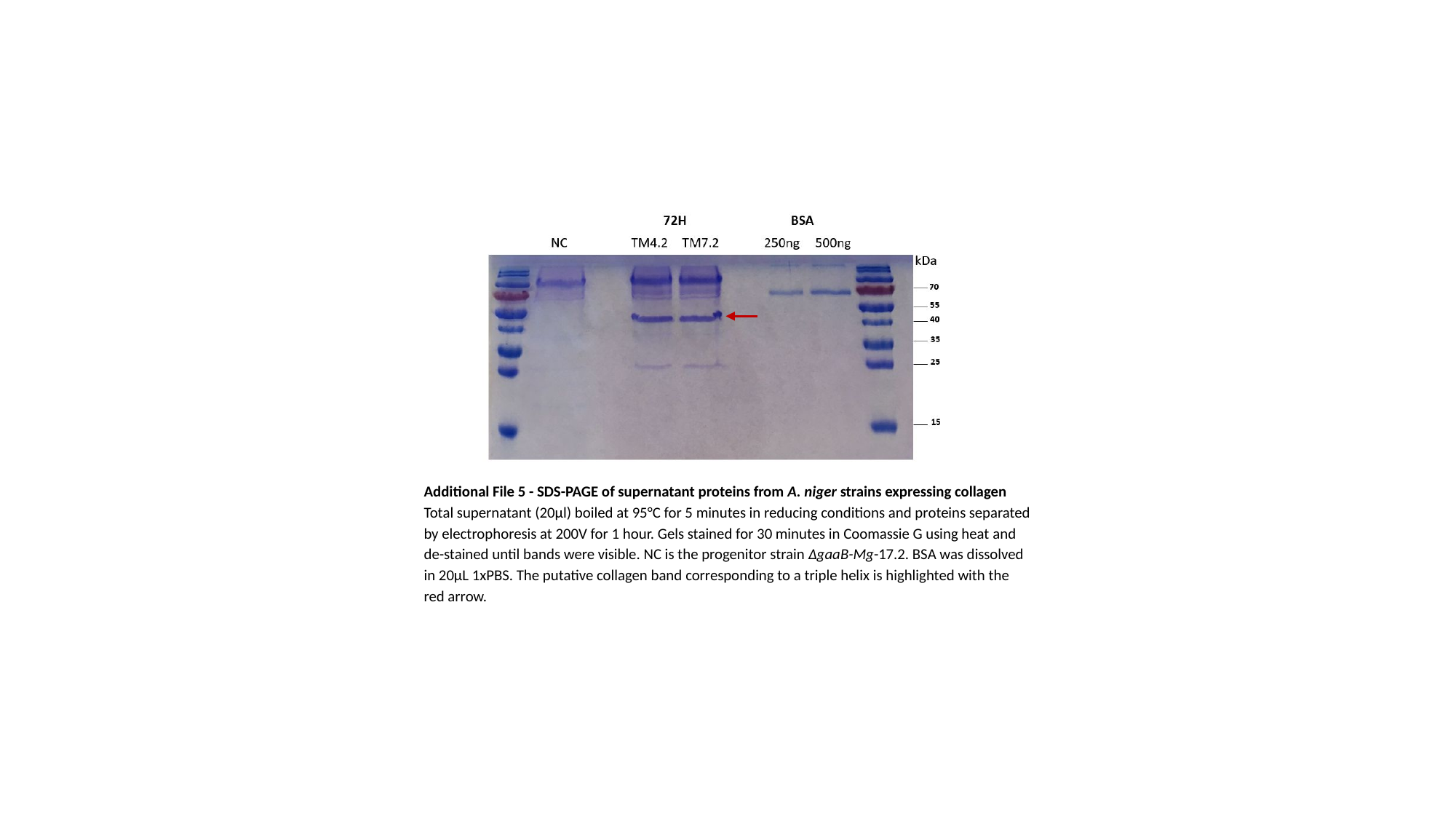

Additional File 5 - SDS-PAGE of supernatant proteins from A. niger strains expressing collagen
Total supernatant (20µl) boiled at 95°C for 5 minutes in reducing conditions and proteins separated by electrophoresis at 200V for 1 hour. Gels stained for 30 minutes in Coomassie G using heat and de-stained until bands were visible. NC is the progenitor strain ΔgaaB-Mg-17.2. BSA was dissolved in 20µL 1xPBS. The putative collagen band corresponding to a triple helix is highlighted with the red arrow.
