# Supplementary material for: The multipurpose cell factory Aspergillus niger can be engineered to produce hydroxylated collagen
Source: Biotechnol Biofuels Bioprod. 2025 Aug 8;18:88. doi: 10.1186/s13068-025-02681-y (PMC12333218; doi:10.1186/s13068-025-02681-y)
Supplement: Supplementary file 6 — Additional file 6. Collagenous fragments obtained from LC-MS data of extracted 17 kDa band from A. niger isolate TM4.2.1. [file 13068_2025_2681_MOESM6_ESM.pptx]

## Slide 1
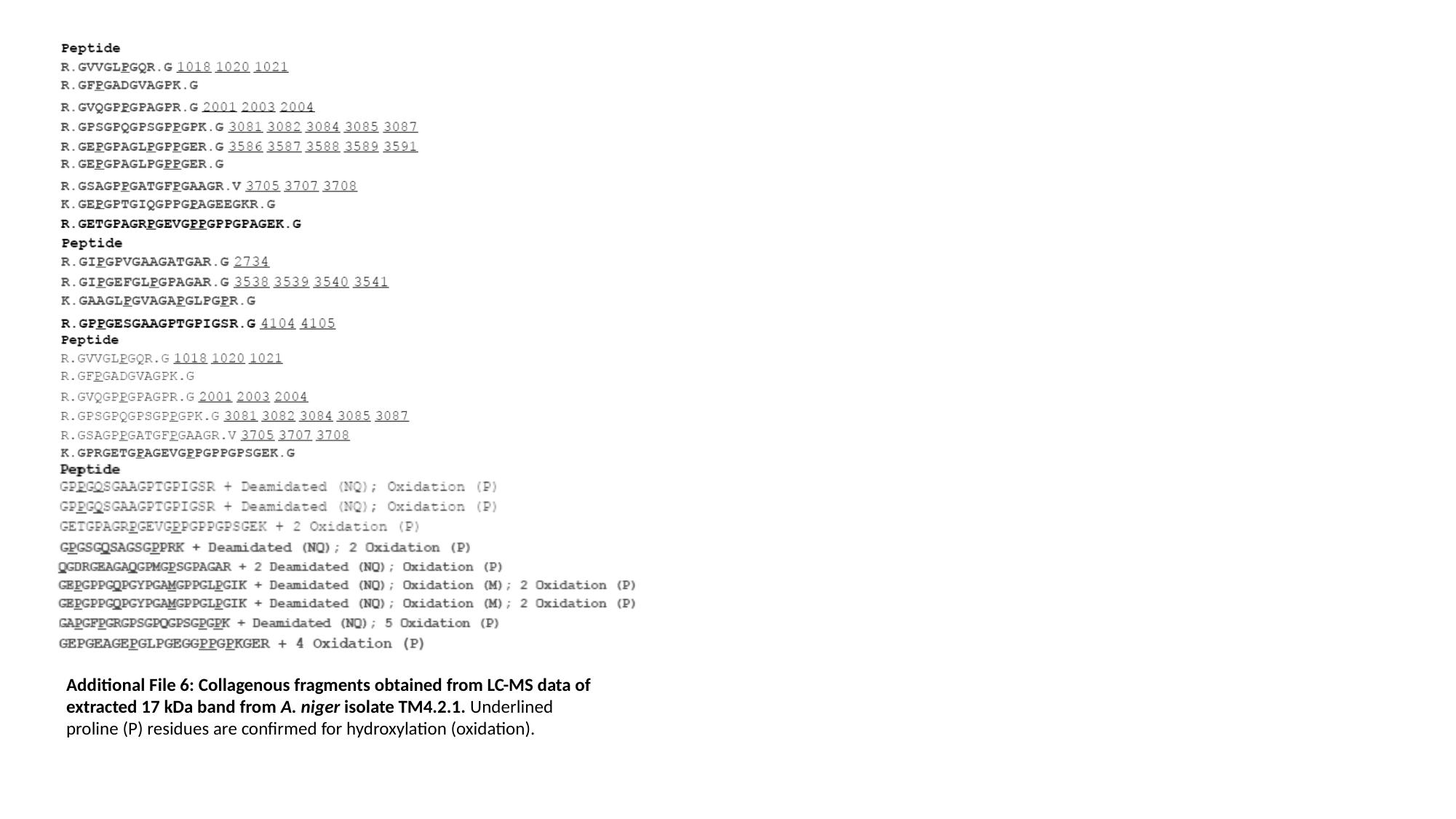

Additional File 6: Collagenous fragments obtained from LC-MS data of extracted 17 kDa band from A. niger isolate TM4.2.1. Underlined proline (P) residues are confirmed for hydroxylation (oxidation).
