# Supplementary material for: The multipurpose cell factory Aspergillus niger can be engineered to produce hydroxylated collagen
Source: Biotechnol Biofuels Bioprod. 2025 Aug 8;18:88. doi: 10.1186/s13068-025-02681-y (PMC12333218; doi:10.1186/s13068-025-02681-y)
Supplement: Supplementary file 7 — Additional file 7. Transcriptomic tables. [file 13068_2025_2681_MOESM7_ESM.pptx]

## Slide 1
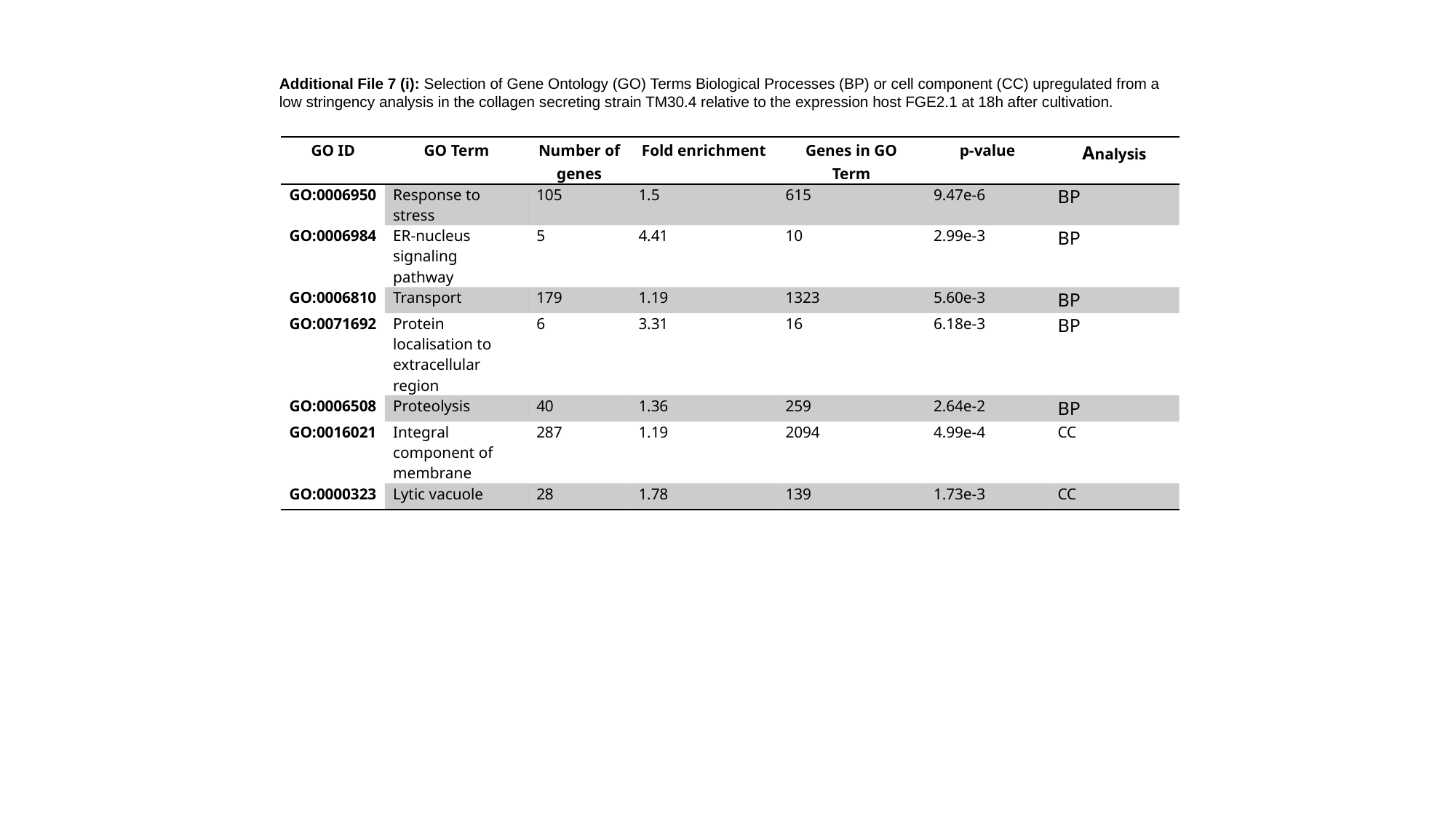

Additional File 7 (i): Selection of Gene Ontology (GO) Terms Biological Processes (BP) or cell component (CC) upregulated from a low stringency analysis in the collagen secreting strain TM30.4 relative to the expression host FGE2.1 at 18h after cultivation.
| GO ID | GO Term | Number of genes | Fold enrichment | Genes in GO Term | p-value | Analysis | |
| --- | --- | --- | --- | --- | --- | --- | --- |
| GO:0006950 | Response to stress | 105 | 1.5 | 615 | 9.47e-6 | BP | |
| GO:0006984 | ER-nucleus signaling pathway | 5 | 4.41 | 10 | 2.99e-3 | BP | |
| GO:0006810 | Transport | 179 | 1.19 | 1323 | 5.60e-3 | BP | |
| GO:0071692 | Protein localisation to extracellular region | 6 | 3.31 | 16 | 6.18e-3 | BP | |
| GO:0006508 | Proteolysis | 40 | 1.36 | 259 | 2.64e-2 | BP | |
| GO:0016021 | Integral component of membrane | 287 | 1.19 | 2094 | 4.99e-4 | CC | |
| GO:0000323 | Lytic vacuole | 28 | 1.78 | 139 | 1.73e-3 | CC | |

## Slide 2
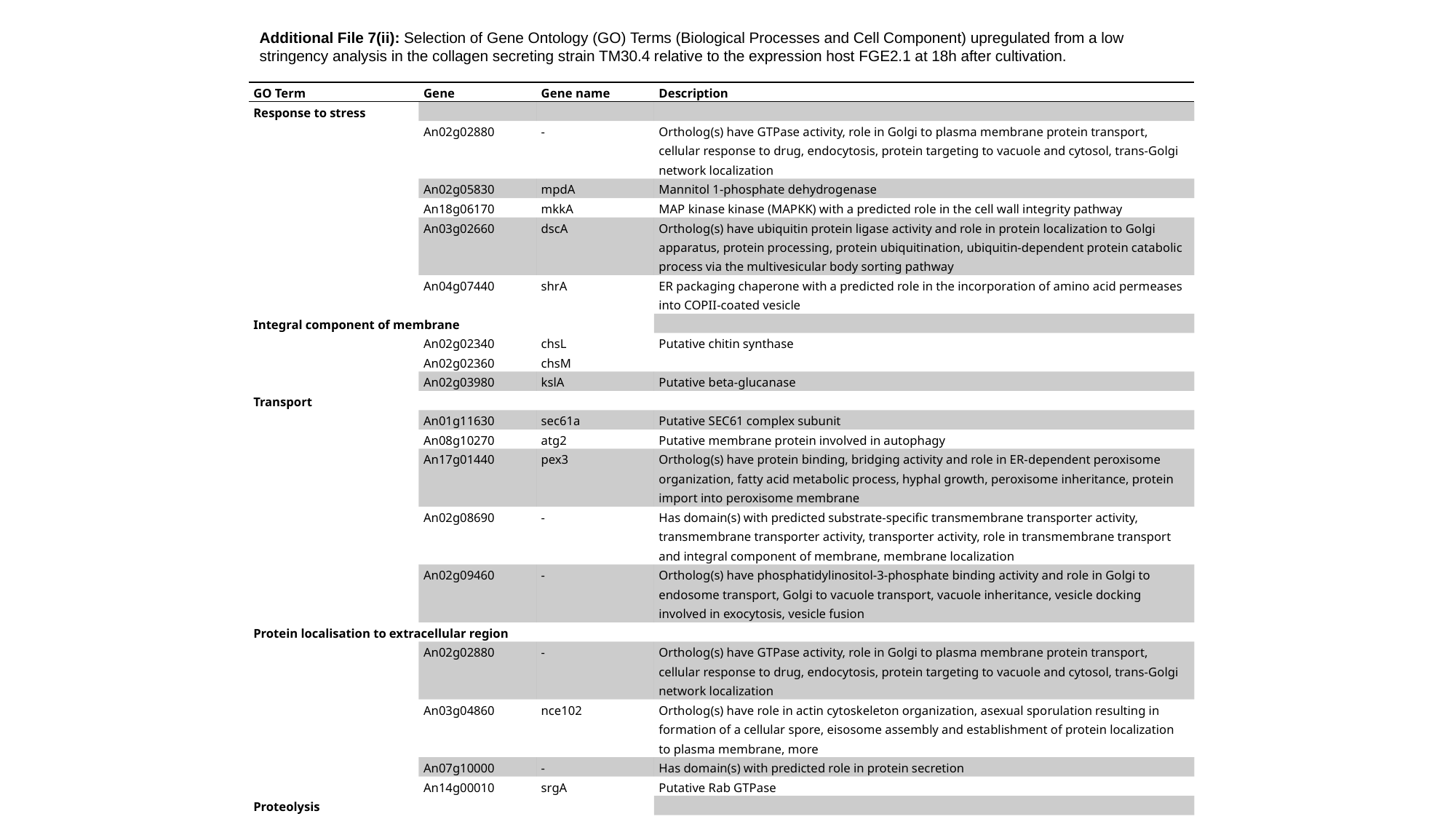

Additional File 7(ii): Selection of Gene Ontology (GO) Terms (Biological Processes and Cell Component) upregulated from a low stringency analysis in the collagen secreting strain TM30.4 relative to the expression host FGE2.1 at 18h after cultivation.
| GO Term | Gene | Gene name | Description |
| --- | --- | --- | --- |
| Response to stress | | | |
| | An02g02880 | - | Ortholog(s) have GTPase activity, role in Golgi to plasma membrane protein transport, cellular response to drug, endocytosis, protein targeting to vacuole and cytosol, trans-Golgi network localization |
| | An02g05830 | mpdA | Mannitol 1-phosphate dehydrogenase |
| | An18g06170 | mkkA | MAP kinase kinase (MAPKK) with a predicted role in the cell wall integrity pathway |
| | An03g02660 | dscA | Ortholog(s) have ubiquitin protein ligase activity and role in protein localization to Golgi apparatus, protein processing, protein ubiquitination, ubiquitin-dependent protein catabolic process via the multivesicular body sorting pathway |
| | An04g07440 | shrA | ER packaging chaperone with a predicted role in the incorporation of amino acid permeases into COPII-coated vesicle |
| Integral component of membrane | | | |
| | An02g02340 An02g02360 | chsL chsM | Putative chitin synthase |
| | An02g03980 | kslA | Putative beta-glucanase |
| Transport | | | |
| | An01g11630 | sec61a | Putative SEC61 complex subunit |
| | An08g10270 | atg2 | Putative membrane protein involved in autophagy |
| | An17g01440 | pex3 | Ortholog(s) have protein binding, bridging activity and role in ER-dependent peroxisome organization, fatty acid metabolic process, hyphal growth, peroxisome inheritance, protein import into peroxisome membrane |
| | An02g08690 | - | Has domain(s) with predicted substrate-specific transmembrane transporter activity, transmembrane transporter activity, transporter activity, role in transmembrane transport and integral component of membrane, membrane localization |
| | An02g09460 | - | Ortholog(s) have phosphatidylinositol-3-phosphate binding activity and role in Golgi to endosome transport, Golgi to vacuole transport, vacuole inheritance, vesicle docking involved in exocytosis, vesicle fusion |
| Protein localisation to extracellular region | | | |
| | An02g02880 | - | Ortholog(s) have GTPase activity, role in Golgi to plasma membrane protein transport, cellular response to drug, endocytosis, protein targeting to vacuole and cytosol, trans-Golgi network localization |
| | An03g04860 | nce102 | Ortholog(s) have role in actin cytoskeleton organization, asexual sporulation resulting in formation of a cellular spore, eisosome assembly and establishment of protein localization to plasma membrane, more |
| | An07g10000 | - | Has domain(s) with predicted role in protein secretion |
| | An14g00010 | srgA | Putative Rab GTPase |
| Proteolysis | | | |
| | An18g06700 | pre7 | 20S CP beta subunit of the proteasome |
| | An08g00430 | kexA | Ortholog(s) have serine-type carboxypeptidase activity, role in apoptotic process, hyphal growth, positive regulation of conidium formation and fungal-type vacuole, trans-Golgi network localization |
| | An07g03880 | pepC | Putative vacuolar serine proteinase |
| | An08g04490 | protA | Secreted lysosomal Pro-Xaa carboxypeptidase |
| | An03g05200 | protF | Carboxypeptidase Y family secreted protease |

## Slide 3
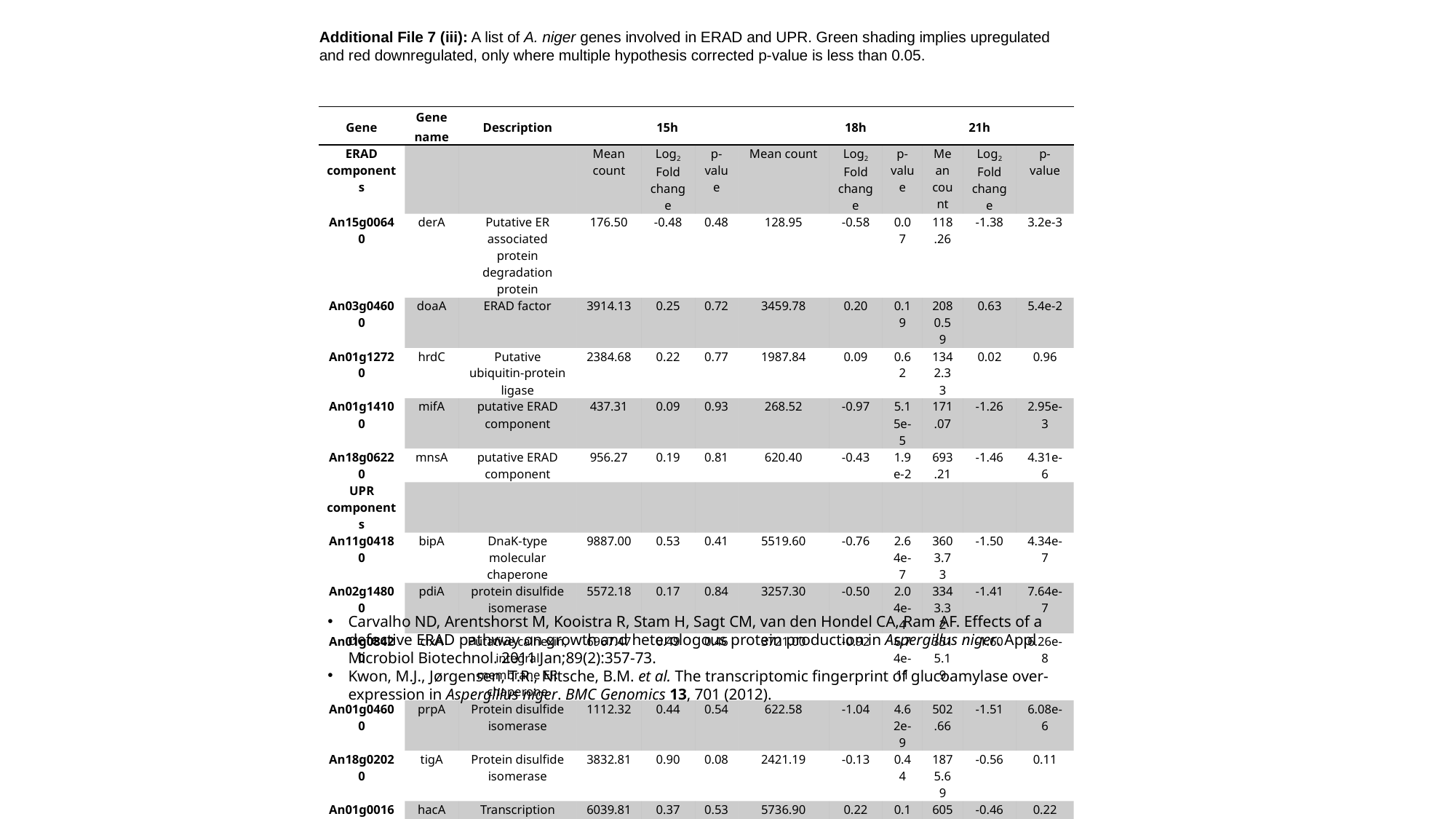

Additional File 7 (iii): A list of A. niger genes involved in ERAD and UPR. Green shading implies upregulated and red downregulated, only where multiple hypothesis corrected p-value is less than 0.05.
| Gene | Gene name | Description | 15h | | | | | 18h | | | | | 21h | | |
| --- | --- | --- | --- | --- | --- | --- | --- | --- | --- | --- | --- | --- | --- | --- | --- |
| ERAD components | | | Mean count | Log2 Fold change | p-value | Mean count | | | Log2 Fold change | p-value | | Mean count | | Log2 Fold change | p-value |
| An15g00640 | derA | Putative ER associated protein degradation protein | 176.50 | -0.48 | 0.48 | 128.95 | | | -0.58 | 0.07 | | 118.26 | | -1.38 | 3.2e-3 |
| An03g04600 | doaA | ERAD factor | 3914.13 | 0.25 | 0.72 | 3459.78 | | | 0.20 | 0.19 | | 2080.59 | | 0.63 | 5.4e-2 |
| An01g12720 | hrdC | Putative ubiquitin-protein ligase | 2384.68 | 0.22 | 0.77 | 1987.84 | | | 0.09 | 0.62 | | 1342.33 | | 0.02 | 0.96 |
| An01g14100 | mifA | putative ERAD component | 437.31 | 0.09 | 0.93 | 268.52 | | | -0.97 | 5.15e-5 | | 171.07 | | -1.26 | 2.95e-3 |
| An18g06220 | mnsA | putative ERAD component | 956.27 | 0.19 | 0.81 | 620.40 | | | -0.43 | 1.9e-2 | | 693.21 | | -1.46 | 4.31e-6 |
| UPR components | | | | | | | | | | | | | | | |
| An11g04180 | bipA | DnaK-type molecular chaperone | 9887.00 | 0.53 | 0.41 | 5519.60 | | | -0.76 | 2.64e-7 | | 3603.73 | | -1.50 | 4.34e-7 |
| An02g14800 | pdiA | protein disulfide isomerase | 5572.18 | 0.17 | 0.84 | 3257.30 | | | -0.50 | 2.04e-4 | | 3343.32 | | -1.41 | 7.64e-7 |
| An01g08420 | clxA | Putative calnexin, integral membrane ER chaperone | 6967.47 | 0.49 | 0.46 | 3721.00 | | | -0.92 | 6.74e-11 | | 3515.19 | | -1.60 | 6.26e-8 |
| An01g04600 | prpA | Protein disulfide isomerase | 1112.32 | 0.44 | 0.54 | 622.58 | | | -1.04 | 4.62e-9 | | 502.66 | | -1.51 | 6.08e-6 |
| An18g02020 | tigA | Protein disulfide isomerase | 3832.81 | 0.90 | 0.08 | 2421.19 | | | -0.13 | 0.44 | | 1875.69 | | -0.56 | 0.11 |
| An01g00160 | hacA | Transcription factor UPR mediator | 6039.81 | 0.37 | 0.53 | 5736.90 | | | 0.22 | 0.13 | | 6059.63 | | -0.46 | 0.22 |
Carvalho ND, Arentshorst M, Kooistra R, Stam H, Sagt CM, van den Hondel CA, Ram AF. Effects of a defective ERAD pathway on growth and heterologous protein production in Aspergillus niger. Appl Microbiol Biotechnol. 2011 Jan;89(2):357-73.
Kwon, M.J., Jørgensen, T.R., Nitsche, B.M. et al. The transcriptomic fingerprint of glucoamylase over-expression in Aspergillus niger. BMC Genomics 13, 701 (2012).

## Slide 4
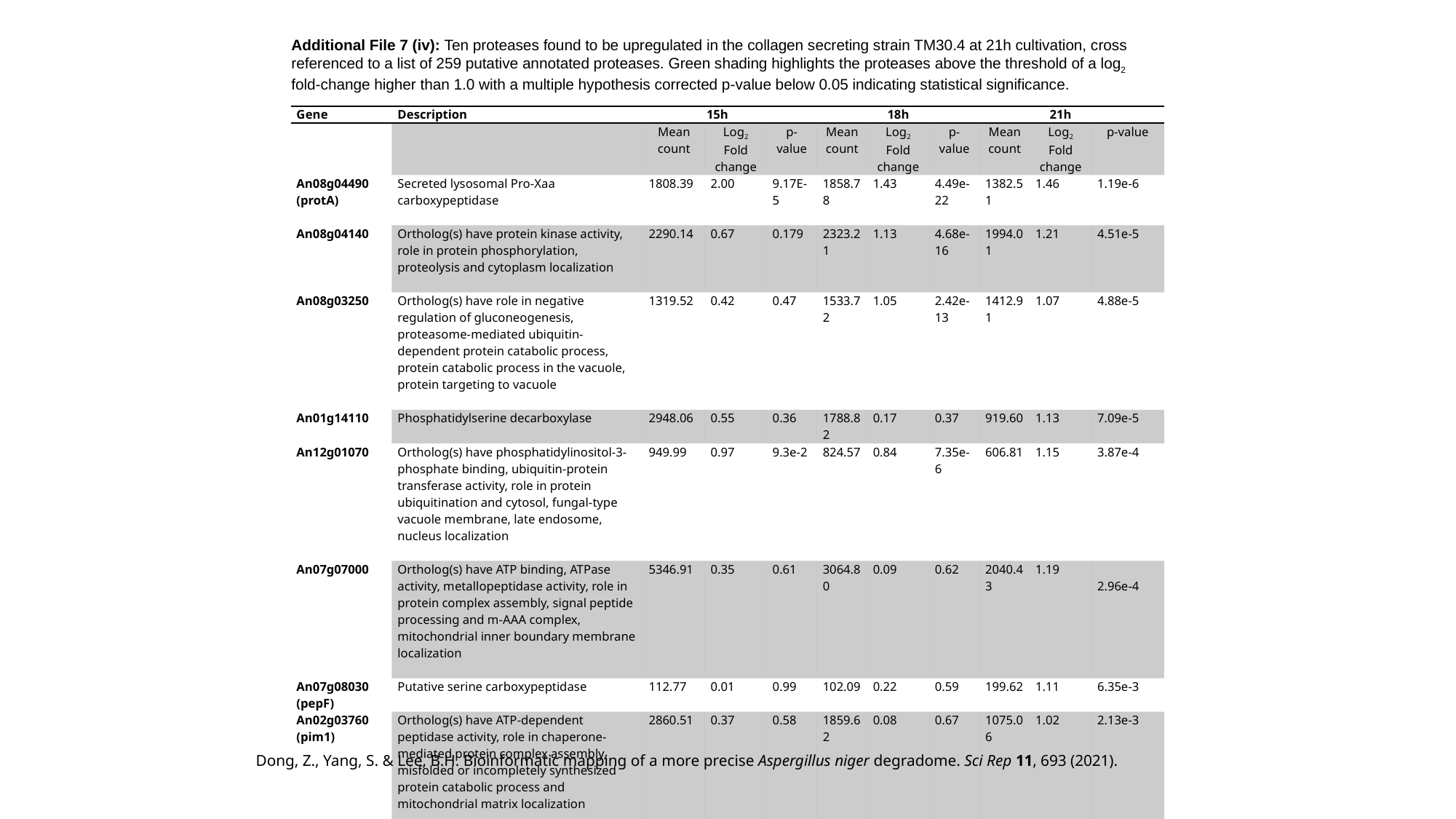

Additional File 7 (iv): Ten proteases found to be upregulated in the collagen secreting strain TM30.4 at 21h cultivation, cross referenced to a list of 259 putative annotated proteases. Green shading highlights the proteases above the threshold of a log2 fold-change higher than 1.0 with a multiple hypothesis corrected p-value below 0.05 indicating statistical significance.
| Gene | Description | 15h | | | | | 18h | | | | | 21h | | | |
| --- | --- | --- | --- | --- | --- | --- | --- | --- | --- | --- | --- | --- | --- | --- | --- |
| | | Mean count | Log2 Fold change | p-value | | Mean count | | Log2 Fold change | p-value | | Mean count | | Log2 Fold change | p-value | |
| An08g04490 (protA) | Secreted lysosomal Pro-Xaa carboxypeptidase | 1808.39 | 2.00 | 9.17E-5 | | 1858.78 | | 1.43 | 4.49e-22 | | 1382.51 | | 1.46 | 1.19e-6 | |
| An08g04140 | Ortholog(s) have protein kinase activity, role in protein phosphorylation, proteolysis and cytoplasm localization | 2290.14 | 0.67 | 0.179 | | 2323.21 | | 1.13 | 4.68e-16 | | 1994.01 | | 1.21 | 4.51e-5 | |
| An08g03250 | Ortholog(s) have role in negative regulation of gluconeogenesis, proteasome-mediated ubiquitin-dependent protein catabolic process, protein catabolic process in the vacuole, protein targeting to vacuole | 1319.52 | 0.42 | 0.47 | | 1533.72 | | 1.05 | 2.42e-13 | | 1412.91 | | 1.07 | 4.88e-5 | |
| An01g14110 | Phosphatidylserine decarboxylase | 2948.06 | 0.55 | 0.36 | | 1788.82 | | 0.17 | 0.37 | | 919.60 | | 1.13 | 7.09e-5 | |
| An12g01070 | Ortholog(s) have phosphatidylinositol-3-phosphate binding, ubiquitin-protein transferase activity, role in protein ubiquitination and cytosol, fungal-type vacuole membrane, late endosome, nucleus localization | 949.99 | 0.97 | 9.3e-2 | | 824.57 | | 0.84 | 7.35e-6 | | 606.81 | | 1.15 | 3.87e-4 | |
| An07g07000 | Ortholog(s) have ATP binding, ATPase activity, metallopeptidase activity, role in protein complex assembly, signal peptide processing and m-AAA complex, mitochondrial inner boundary membrane localization | 5346.91 | 0.35 | 0.61 | | 3064.80 | | 0.09 | 0.62 | | 2040.43 | | 1.19 | 2.96e-4 | |
| An07g08030 (pepF) | Putative serine carboxypeptidase | 112.77 | 0.01 | 0.99 | | 102.09 | | 0.22 | 0.59 | | 199.62 | | 1.11 | 6.35e-3 | |
| An02g03760 (pim1) | Ortholog(s) have ATP-dependent peptidase activity, role in chaperone-mediated protein complex assembly, misfolded or incompletely synthesized protein catabolic process and mitochondrial matrix localization | 2860.51 | 0.37 | 0.58 | | 1859.62 | | 0.08 | 0.67 | | 1075.06 | | 1.02 | 2.13e-3 | |
| An17g00760 | Ortholog(s) have extracellular region localization | 248.53 | 0.92 | 8.9e-2 | | 377.10 | | 1.84 | 1.30e-18 | | 385.85 | | 1.98 | 9.95e-10 | |
| An09g04470 (casB) | Ortholog(s) have calcium-dependent cysteine-type endopeptidase activity | 1049.94 | 1.02 | 6.5e-2 | | 915.01 | | 0.71 | 1.17e-5 | | 687.27 | | 1.05 | 2.88e-3 | |
Dong, Z., Yang, S. & Lee, B.H. Bioinformatic mapping of a more precise Aspergillus niger degradome. Sci Rep 11, 693 (2021).
