# Supplementary material for: The multipurpose cell factory Aspergillus niger can be engineered to produce hydroxylated collagen
Source: Biotechnol Biofuels Bioprod. 2025 Aug 8;18:88. doi: 10.1186/s13068-025-02681-y (PMC12333218; doi:10.1186/s13068-025-02681-y)
Supplement: Supplementary file 8 — Additional file 8. A. niger strains expressing collagen III under the tef1 promoter secrete the protein into the supernatant. [file 13068_2025_2681_MOESM8_ESM.pptx]

## Slide 1
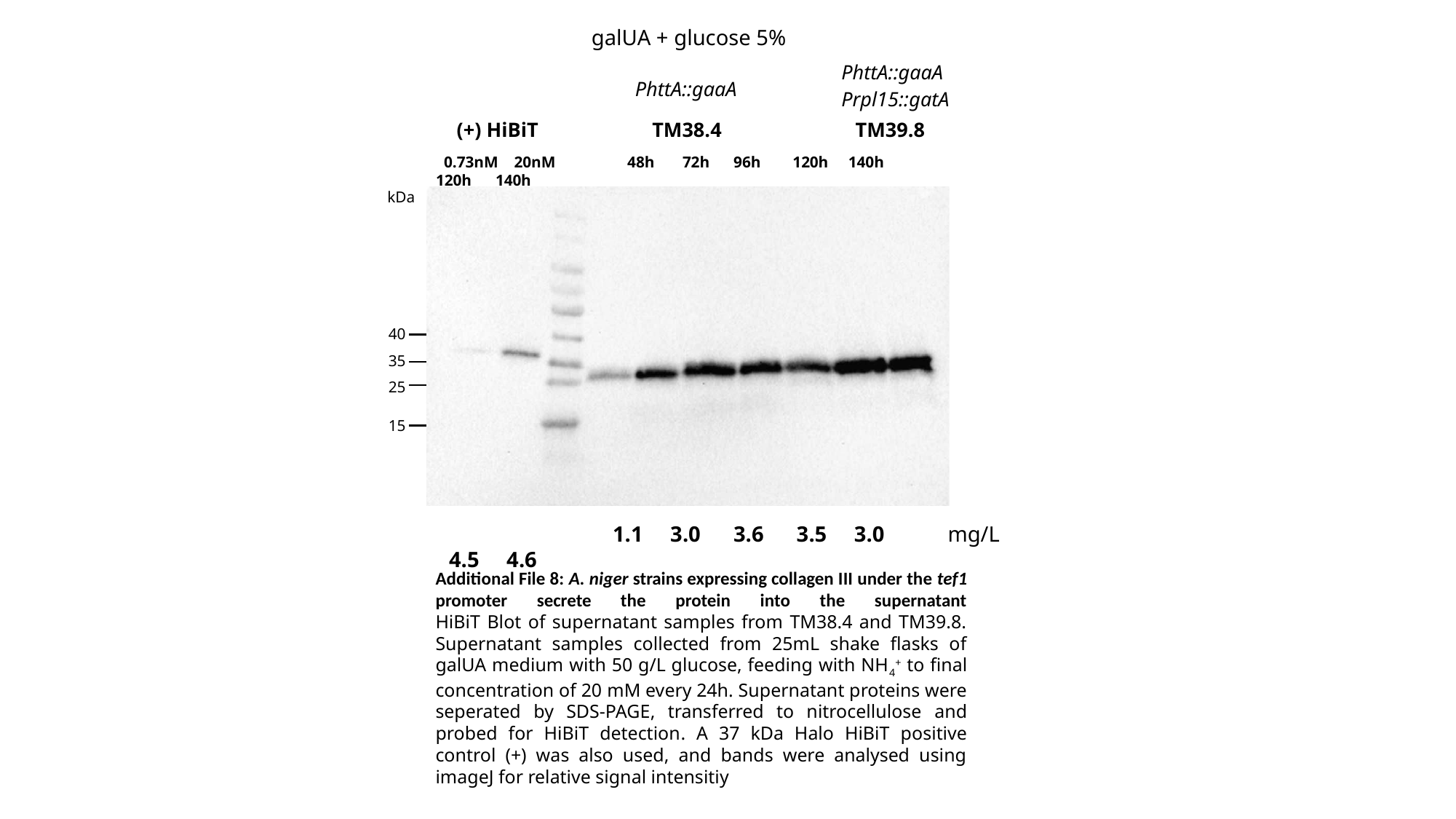

galUA + glucose 5%
PhttA::gaaA
Prpl15::gatA
PhttA::gaaA
(+) HiBiT
TM38.4
TM39.8
 0.73nM 20nM 48h 72h 96h 120h 140h 120h 140h
 1.1 3.0 3.6 3.5 3.0 4.5 4.6
mg/L
kDa
40
35
25
15
Additional File 8: A. niger strains expressing collagen III under the tef1 promoter secrete the protein into the supernatant
HiBiT Blot of supernatant samples from TM38.4 and TM39.8. Supernatant samples collected from 25mL shake flasks of galUA medium with 50 g/L glucose, feeding with NH4+ to final concentration of 20 mM every 24h. Supernatant proteins were seperated by SDS-PAGE, transferred to nitrocellulose and probed for HiBiT detection. A 37 kDa Halo HiBiT positive control (+) was also used, and bands were analysed using imageJ for relative signal intensitiy
