# Supplementary material for: The multipurpose cell factory Aspergillus niger can be engineered to produce hydroxylated collagen
Source: Biotechnol Biofuels Bioprod. 2025 Aug 8;18:88. doi: 10.1186/s13068-025-02681-y (PMC12333218; doi:10.1186/s13068-025-02681-y)
Supplement: Supplementary file 9 — Additional file 9. A. niger strains secrete HiBiT tagged collagen III with supernatant stability up to 140 h. [file 13068_2025_2681_MOESM9_ESM.pptx]

## Slide 1
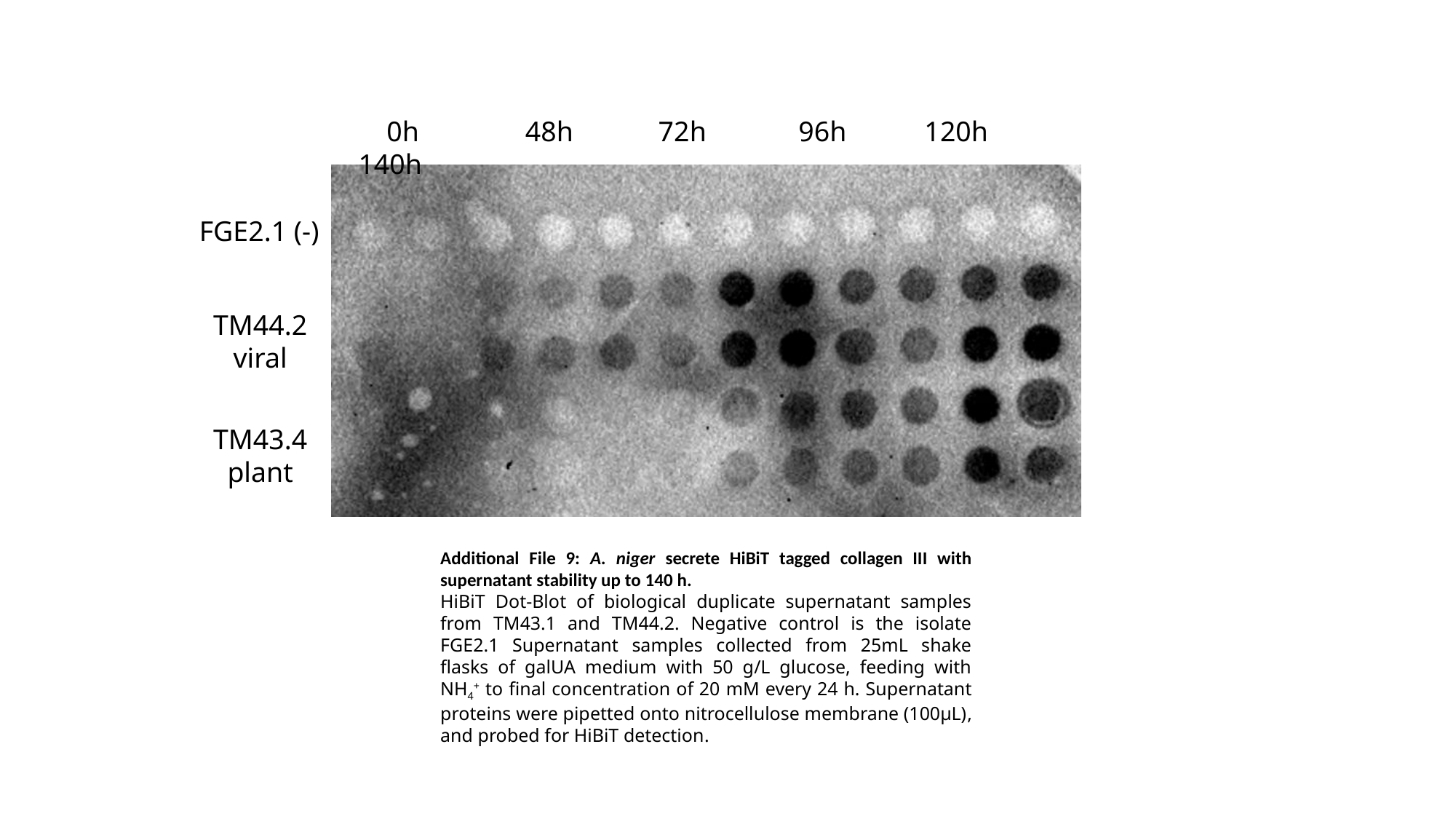

0h 48h 72h 96h 120h 140h
FGE2.1 (-)
TM44.2
viral
TM43.4
plant
Additional File 9: A. niger secrete HiBiT tagged collagen III with supernatant stability up to 140 h.
HiBiT Dot-Blot of biological duplicate supernatant samples from TM43.1 and TM44.2. Negative control is the isolate FGE2.1 Supernatant samples collected from 25mL shake flasks of galUA medium with 50 g/L glucose, feeding with NH4+ to final concentration of 20 mM every 24 h. Supernatant proteins were pipetted onto nitrocellulose membrane (100µL), and probed for HiBiT detection.
