# Supplementary material for: The multipurpose cell factory Aspergillus niger can be engineered to produce hydroxylated collagen
Source: Biotechnol Biofuels Bioprod. 2025 Aug 8;18:88. doi: 10.1186/s13068-025-02681-y (PMC12333218; doi:10.1186/s13068-025-02681-y)
Supplement: Supplementary file 10 — Additional file 10. Schematic of genetic engineering steps to construct the recombinant collagen secreting isolate A. niger strain TM44.2 from the progenitor strain ∆gaaB. [file 13068_2025_2681_MOESM10_ESM.pptx]

## Slide 1
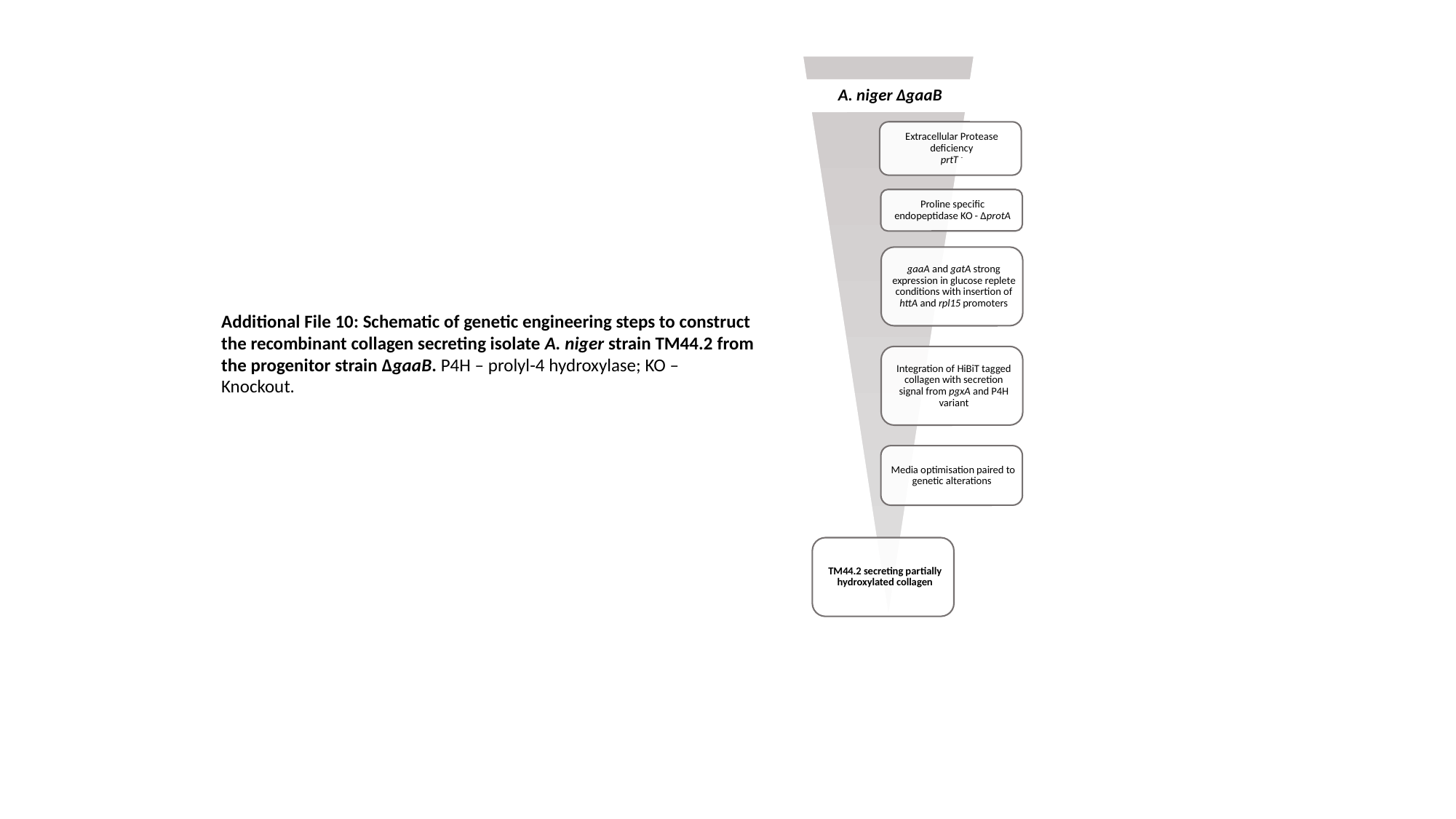

A. niger ∆gaaB
Additional File 10: Schematic of genetic engineering steps to construct the recombinant collagen secreting isolate A. niger strain TM44.2 from the progenitor strain ∆gaaB. P4H – prolyl-4 hydroxylase; KO – Knockout.
