# Supplementary material for: The multipurpose cell factory Aspergillus niger can be engineered to produce hydroxylated collagen
Source: Biotechnol Biofuels Bioprod. 2025 Aug 8;18:88. doi: 10.1186/s13068-025-02681-y (PMC12333218; doi:10.1186/s13068-025-02681-y)
Supplement: Supplementary file 11 — Additional file 11. qPCR results from strains expressing collagen III. [file 13068_2025_2681_MOESM11_ESM.pptx]

## Slide 1
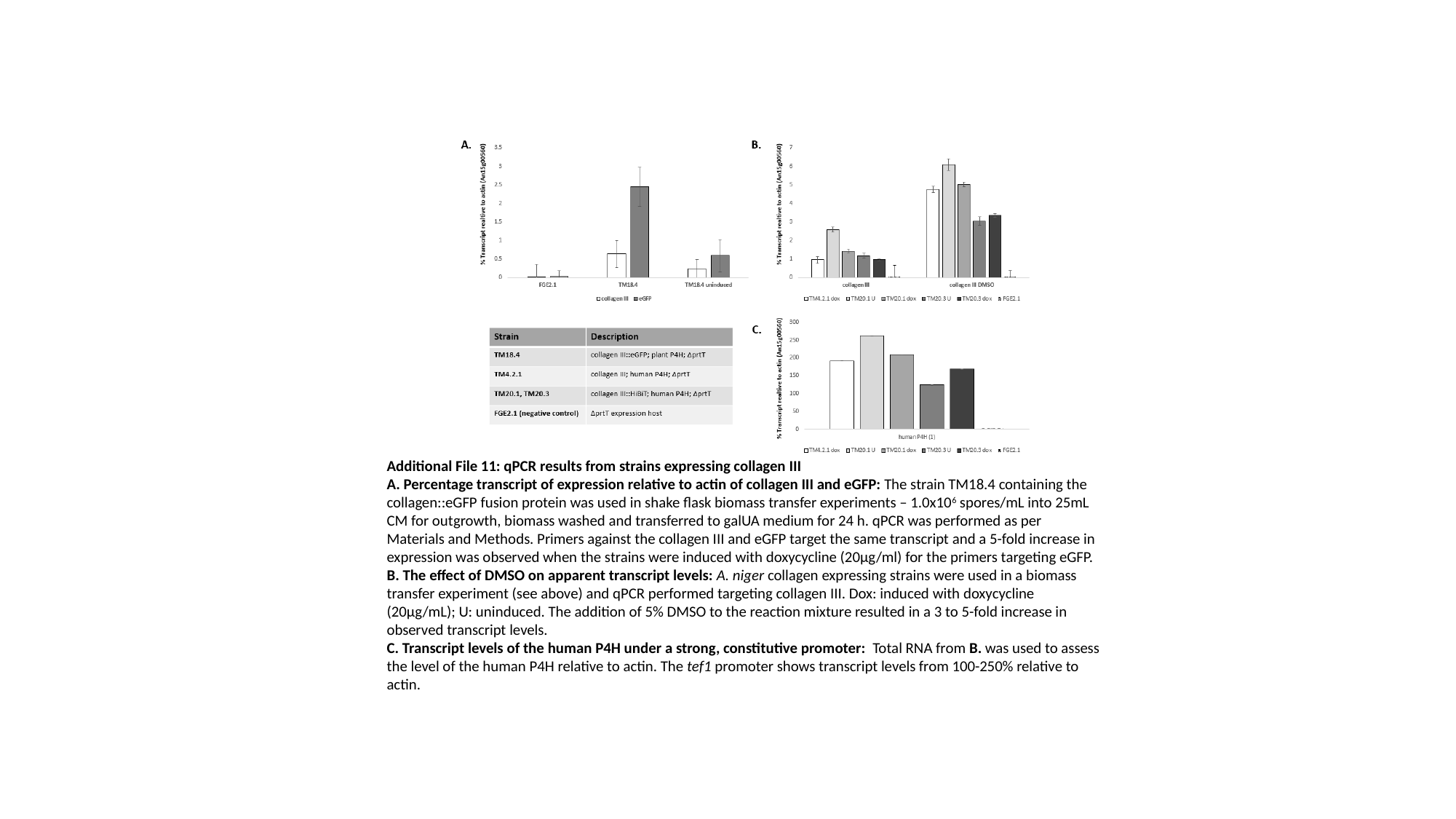

Additional File 11: qPCR results from strains expressing collagen III
A. Percentage transcript of expression relative to actin of collagen III and eGFP: The strain TM18.4 containing the collagen::eGFP fusion protein was used in shake flask biomass transfer experiments – 1.0x10^6^ spores/mL into 25mL CM for outgrowth, biomass washed and transferred to galUA medium for 24 h. qPCR was performed as per Materials and Methods. Primers against the collagen III and eGFP target the same transcript and a 5-fold increase in expression was observed when the strains were induced with doxycycline (20µg/ml) for the primers targeting eGFP.
B. The effect of DMSO on apparent transcript levels: A. niger collagen expressing strains were used in a biomass transfer experiment (see above) and qPCR performed targeting collagen III. Dox: induced with doxycycline (20µg/mL); U: uninduced. The addition of 5% DMSO to the reaction mixture resulted in a 3 to 5-fold increase in observed transcript levels.
C. Transcript levels of the human P4H under a strong, constitutive promoter: Total RNA from B. was used to assess the level of the human P4H relative to actin. The tef1 promoter shows transcript levels from 100-250% relative to actin.
